# Differential Radiomodulatory Effects of Sodium Aminodihydrophthalazinedione (Tameron^®^) on Normal and Cancer Cells Cultures: Antioxidant Activity, DNA Damage Response, and Transcriptomic Profiling

**DOI:** 10.3390/ijms27125272

**Published:** 2026-06-10

**Authors:** Artem Ermakov, Elena Tsarkova, Olga Ermakova, Olga Antonova, Olga Kochetkova, Danil Kolmanovich, Anastasia Kolotova, Edward Evdokimovskii, Artem Blagodatski, Anton Popov

**Affiliations:** 1 Institute of Theoretical and Experimental Biophysics of the Russian Academy of Sciences, 3 Institutskaya Str., Pushchino 142290, Russia; ermakova.iteb@iteb.ru (O.E.); olga.antonova.iteb@gmail.com (O.A.); o.y.kochetkova@gmail.com (O.K.); kdd100996@mail.ru (D.K.); anas.kolotowa2010@ya.ru (A.K.); onletaet@gmail.com (E.E.); bswin2000@gmail.com (A.B.); antonpopovleonid@gmail.com (A.P.); 2 ANO Engineering Physics Institute, Bolshoi Udarny Pereulok, 1A, Bldg. 1, Serpuhov 142210, Russia; elenaa_kulakova@mail.ru; 3 LLC Nanoporus, 2nd Moskovskaya Street, 6, Room 5, Serpuhov 142210, Russia; 4 Scientific and Educational Center, Federal State University of Education, 24 ul. Very Voloshinoi, Moscow 141014, Russia

**Keywords:** mesenchymal stem cells, osteosarcoma, Tameron, antioxidant, radioprotection, luminol, DNA double-strand breaks, transcriptomics, nanopore sequencing, radiotherapy, reactive oxygen species

## Abstract

Radiotherapy is one of the most effective methods of cancer treatment. New, more effective, and safer radiotherapy methods can be developed thanks to selective radioprotectors. In our study, we investigated the antioxidant and radiomodulatory activity of sodium aminodihydrophthalazinedione (the drug Tameron^®^) on human mesenchymal stem cells (MSCs) and human osteosarcoma cells of the MNNG/Hos line in vitro. We have shown that sodium aminodihydrophthalazinedione effectively scavenged radiation-induced hydrogen peroxide in aqueous solution in a concentration-dependent manner after X-ray irradiation. We also showed that sodium aminodihydrophthalazinedione (0.25 mM and above) effectively protects human MSCs from the damaging effects of X-rays, reducing the level of intracellular ROS and the number of apoptotic cells after irradiation, enhancing the restoration of double-stranded DNA breaks and clonogenic activity. Meanwhile, the effect of sodium aminodihydrophthalazinedione on human osteosarcoma MNNG/Hos cells was different: it increased the number of apoptotic cells and reduced the rate of repair of double-stranded DNA breaks. Transcriptomic studies on both cell culture types using nanopore sequencing technology after X-ray irradiation and sodium aminodihydrophthalazinedione pretreatment revealed a significant level of modulation of key genes responsible for DNA repair, antioxidant activity, and genome stability. Our data show that sodium aminodihydrophthalazinedione may be a promising therapeutic agent for modulating the cellular effects of radiation exposure.

## 1. Introduction

The problem of preventing radiation damage and adverse reactions accompanying radiation therapy for malignant neoplasms is still relevant. Increasing the effectiveness of radiation therapy involves the use of various methods and approaches that protect healthy tissues surrounding the tumor while maintaining the radiosensitivity of the tumor tissue. One of the most widely used methods of such protection is the use of selective radioprotectors capable of acting at various stages of the development of radiation-induced damage to cells and tissues. Radioprotectors are usually prescribed before a radiation therapy session in order to prevent or mitigate the body’s reactions to radiation. To date, there are several classes of substances that have pronounced radioprotective properties and are used in clinical practice. The most well-known chemical radioprotector is the prodrug amifostine (WR2721), a thiolphosphate compound that, after entering the cell, decomposes under the action of alkaline phosphatase into free aminothiols capable of forming disulfide bonds, providing a pronounced radioprotective effect [1]. The selectivity of the action of amifostine is based on the different activity and concentration of alkaline phosphatase in normal and cancer cells [2]. However, we should note that its therapeutic use is limited due to its high toxicity, strictly regulated treatment time (15–30 min before radiation), and difficulties with administration (the drug requires intravenous administration) [3]. Natural products with antioxidant activity, such as flavonoids, phenolic acids, lycopene, alkaloids, phytohormones, etc., are considered preferred radioprotective agents due to their lower toxicity and higher availability [4]. In particular, troxerutin has been shown to be able to protect the thymus of irradiated mice by inhibiting thymocyte apoptosis after irradiation. Troxerutin blocked the radiation-induced activation of the PTEN-JNK pathway, thereby promoting cell survival [5]. Curcumin has been found to have a protective effect on radiation-induced cataract models in rats. Monthly intake of the drug by intragastric intubation reduced the risk of cataract development by 60%, providing high levels of antioxidant enzymes and low levels of ROS and malondialdehyde after irradiation [6]. Sesamol is a phenolic natural product of plant origin that has a radioprotective effect on mice after irradiation at a dose of 7.5 g. It was administered intraperitoneally and increased the survival rate of animals after irradiation to 100% of the total. Other positive effects of sesamol included: reduction in cytogenetic damage in the bone marrow, prevention of lipid peroxidation, suppression of pro-apoptotic pathways p53 and Bax, and stimulation of crypt cells of the gastrointestinal tract [7]. To date, several immunomodulators based on natural or synthetic antioxidants have been clinically tested as radioprotectors. Thus, it has been shown that the new immunomodulator AS101 (trichloro-(0,0′dioxyethylene) ammonium tellurate), when administered orally, restores hematopoiesis after sublethal doses of cyclophosphamide or lethal doses of X-ray irradiation [8]. At the same time, of all the listed drugs, only amifostine is used in clinical practice, which must be administered to patients in high concentrations, which limits the possibilities for widespread safety of radiation use and reducing the side effects of radiotherapy in general. Thus, there is a need to develop new radioprotective compounds that are effective in low concentrations.

Here, we investigated the radioprotective effect of sodium aminodihydrophthalazinedione (monosodium luminol). It is approved for clinical use in Russia under the trade names Galavit^®^ (Registration Certificate No. P N000088/02) and Tameron^®^ (Registration Certificate No. ЛП-006597). The compound has additionally received regulatory approval in several European countries and is currently under clinical investigation in the United States (Bach Pharma, Inc., North Andover, MA, U.S.A. GVT^®^) for the treatment of neurodegenerative disorders. However, it has not yet received approval from the FDA or EMA centralized authorization. To date, clinical data on its use in oncology and radiation medicine remain limited; namely, Tameron^®^ is currently used only as an immunomodulator in concomitant drug treatment of cancer [9]. It is well known that sodium aminodihydrophthalazinedione (also called sodium luminol) has antioxidant, neuroprotective, anti-inflammatory, and antiviral properties [10,11,12,13,14]. Thus, we demonstrated the possibilities of using sodium aminodihydrophthalazinedione as a selective radioprotective agent using cultures of normal and tumor cells and also identified the molecular mechanisms of its biological activity under ionizing radiation exposure. We hypothesized that, given its known antioxidant and immunomodulatory properties, sodium aminodihydrophthalazinedione may exert differential effects on normal and malignant cells under radiation exposure, owing to their distinct redox environments and proliferative states. Human mesenchymal stem cells were selected as a model of normal radiosensitive tissue, as they represent a clinically relevant cell population at risk of radiation damage during therapy. The MNNG/Hos osteosarcoma line was chosen as a representative bone tumor model, allowing direct comparison within the same tissue context. We analyzed the antioxidant and radioprotective properties of sodium aminodihydrophthalazinedione in aqueous solution and on cultures of normal (human mesenchymal stem cells) and cancer (human osteosarcoma line MNNG/Hos) cells after high-dose X-ray irradiation.

## 2. Results and Discussion

### 2.1. Tameron^®^ Reduces the Generation of Hydrogen Peroxide After X-Ray Irradiation of Aqueous Solutions

**Figure 1:**
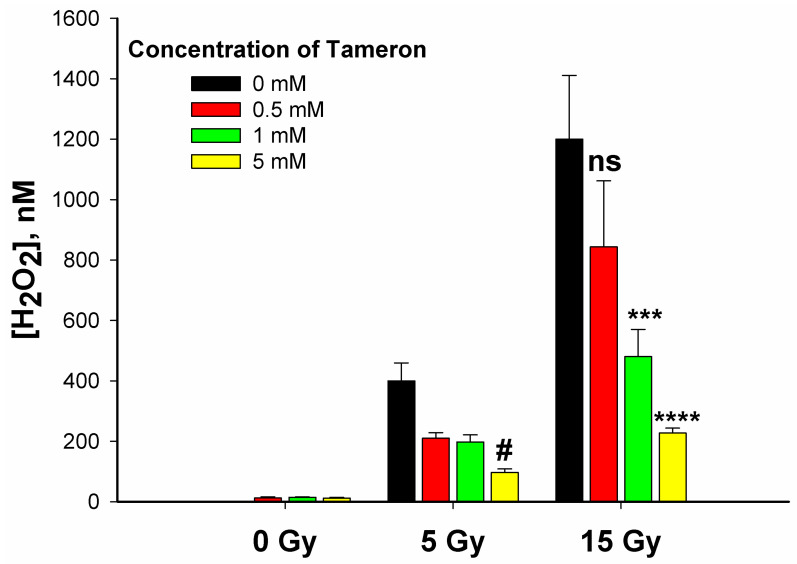
Concentration dependences of the formation of hydrogen peroxide under the action of X-ray irradiation in a buffer solution containing various concentrations of Tameron^®^ (0.5–5 mM). The mean values of three independent experiments and their standard errors are given. The statistical significance of differences between the values in experimental groups was determined using one-way ANOVA. Differences were considered statistically significant at *p* < 0.05 (#) vs. 0.5 mM at 5 Gy, *p* < 0.001 (***) vs. 15 Gy, and *p* < 0.0001 (****) vs. 15 Gy.

The interaction of ionizing radiation with aqueous solutions leads to processes that trigger a cascade of reactions with the formation of short-lived radicals and reactive oxygen species [15]. Hydroxyl radical and hydrogen peroxide are the key damaging factors in the cell after exposure to ionizing radiation [16]. The hydroxyl radical has the properties of a powerful oxidizing agent, reacting with most organic molecules, including proteins, nucleic acids, and lipids. At the same time, the hydroxyl radical has a short lifetime and quickly enters into an oxidation reaction [17]. Hydrogen peroxide is a dismutation product of superoxide radicals, which has a fairly long lifetime (up to several seconds), which allows it to diffuse over distances of up to several microns and damage various cellular structures [18]. In this regard, we studied the level of hydrogen peroxide formation in aqueous solutions of Tameron^®^ (0.5–5 mM) after their irradiation with X-rays (Figure 1). It has been shown that an irradiation dose of 15 Gy leads to the formation of about 1 μM of hydrogen peroxide. Aqueous solutions containing Tameron^®^ at concentrations of 0.5, 1, and 5 mM reduce the level of hydrogen peroxide to 843 ± 218, 480 ± 89, and 227 ± 16 nM, respectively. Such a significant reduction in hydrogen peroxide levels confirms the ability of Tameron^®^ to effectively decompose hydrogen peroxide, which confirms its direct antioxidant activity. Thus, we can speak about the pronounced antioxidant properties of Tameron^®^ and its ability to effectively prevent the radiation-induced formation of hydrogen peroxide in aqueous solutions.

### 2.2. Tameron^®^ Exhibits Selective Cytotoxicity upon X-Ray Irradiation In Vitro

**Figure 2:**
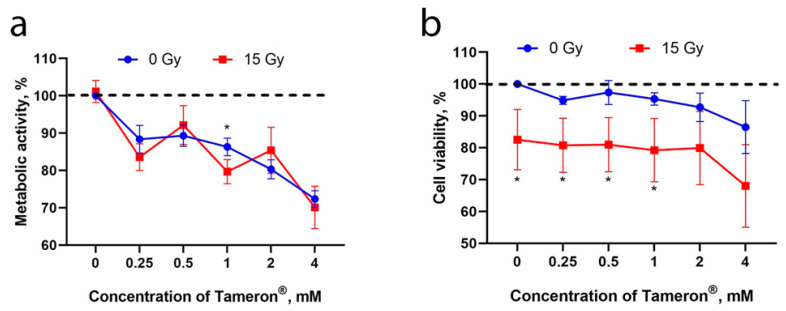
Viability analysis of hMSCs (**a**) and MNNG/Hos osteosarcoma cells (**b**) after incubation with various concentrations of Tameron^®^ (0.25–4 mM) 72 h after X-ray irradiation at a dose of 15 Gy. Data presented as mean± standard deviation (SD). * *p* < 0.05 (*) value was estimated using one-way ANOVA (*n* = 3).

Confirmation of the pronounced dose-dependent antioxidant activity of Tameron^®^ after irradiation with high doses of X-rays suggests its high efficiency in the in vitro irradiation of cell cultures, which was carried out on normal and cancer cell cultures in vitro. The MTT assay method was used to analyze the level of metabolic activity of cell cultures after irradiation in the presence of Tameron^®^, which revealed that human MSCs after irradiation at a dose of 15 Gy in the presence of Tameron^®^ reduce their metabolic activity with increasing concentration, while under irradiation conditions, a similar trend is observed (Figure 2). Human osteosarcoma cell cultures showed a less pronounced trend towards a decrease in the level of metabolic activity with an increase in the concentration of Tameron^®^.

**Figure 3:**
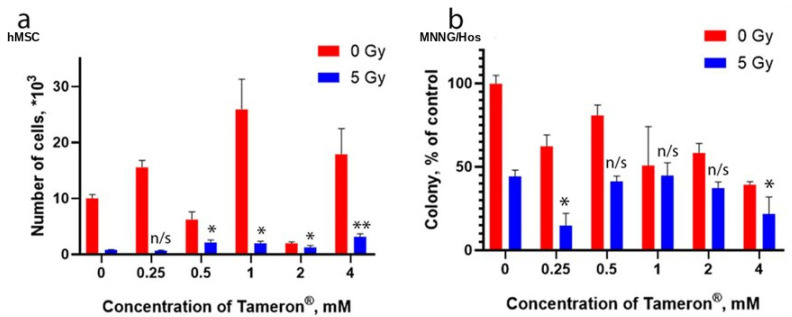
Cell number count estimated by the MTT assay of hMSC (**a**) and clonogenic analysis of the MNNG/Hos human osteosarcoma culture (**b**) after X-ray irradiation in the presence of various concentrations of Tameron^®^ (0.25–4 mM, 8-day incubation). HMSC numbers were determined by a pre-validated MTT-based calibration curve, in which optical density values were converted to cell counts using a standard curve constructed by seeding known cell densities (0.5–20.0 × 10^3^ cells/well). Data presented as mean± standard deviation (SD), and the *p*-value was estimated using one-way ANOVA (n = 5); * *p* ≤ 0.05, ** *p* ≤ 0.001, n/s—not significant. The values of statistical significance are indicated in comparison with the irradiated control.

Meanwhile, it should be taken into account that the MTT assay has its drawbacks when analyzing the viability of cells exposed to high doses of ionizing radiation [19]. In this connection, we additionally conducted an analysis of viability by the analysis of clonogenic activity, which consists of the ability of single cells to form cell colonies after exposure to ionizing radiation [20]. Loss of reproductive integrity and inability to proliferate are the most characteristic signs of the action of a cytotoxic agent or ionizing radiation. A cell that retains the ability to synthesize proteins and DNA and undergoes one or two mitoses but is not able to produce a large number of offspring in the form of colonies is considered dead. For hMSCs, which do not form colonies per se, we have just counted the cell number in the case of this cell line, by means of a previously calibrated MTT assay. Here we have shown that Tameron^®^, in a dose-dependent manner, is able to increase the number of hMSC after irradiation at a dose of 5 Gy (Figure 3). It can be seen from the diagram that the pretreatment of cells with Tameron^®^ at concentrations of 0.5, 1, 2, and 4 mM increases the number of cells compared to the control (untreated with Tameron^®^) group. Pretreatment of human osteosarcoma cells of the MNNG/Hos line with Tameron^®^ at various concentrations also slightly affected the number of colonies, while irradiation of cells with X-rays at a dose of 5 Gy reduced the number of colonies by 20%, and the pretreatment of osteosarcoma cells with various concentrations of Tameron^®^ did not protect them, and the number of colonies was lower at all concentrations tested. It should be noted that with increasing concentration of the drug, the difference in the number of colonies increased, so at a concentration of 4 mM, the drug acted as a sensitizer. Thus, we can conclude that Tameron can selectively delay the death of tumor cells while protecting normal cells. It is known that tumor cells have a higher proliferation rate, as well as an altered intracellular redox status, which significantly distinguishes them from normal cells, including hMSCs. Given the fact that Tameron^®^ effectively penetrates the cell and can directly affect the cell’s signaling pathways, as well as modulate its redox homeostasis, we can assume a selective effect on their survival under ionizing radiation exposure.

The apparent discrepancy between the results of the MTT assay and direct cell counting warrants explicit discussion. In the MTT assay, no significant reduction in metabolic activity was observed in hMSCs following irradiation at 15 Gy within the 72 h observation window. This is consistent with the well-documented phenomenon of radiation-induced cellular senescence, in which cells retain mitochondrial dehydrogenase activity and remain metabolically active despite having permanently lost proliferative capacity [21,22,23]. Indeed, morphological signs of senescence—nuclear enlargement and increased cytoplasmic volume—were clearly observed in irradiated hMSC cultures, supporting this interpretation. By contrast, direct cell counting at 5 Gy over a longer observation period reflects the cumulative loss of proliferating cells, capturing radiation-induced growth arrest more sensitively than short-term metabolic readouts. Thus, the two methods address fundamentally different endpoints: MTT measures short-term metabolic viability, while cell counting reflects net proliferative capacity over time. These two parameters are not expected to correlate under conditions of radiation-induced senescence, and their apparent inconsistency is therefore not a contradiction but rather a reflection of the complexity of the cellular radiation response. This distinction also underscores why the clonogenic assay, as the most direct measure of long-term reproductive integrity, remains the gold standard for assessing radioprotective efficacy in vitro.

### 2.3. Tameron^®^ Does Not Cause Cell Death and Shows a Radioprotective Effect on hMSC, While Causing Cell Death in MNNG/Hos

**Figure 4:**
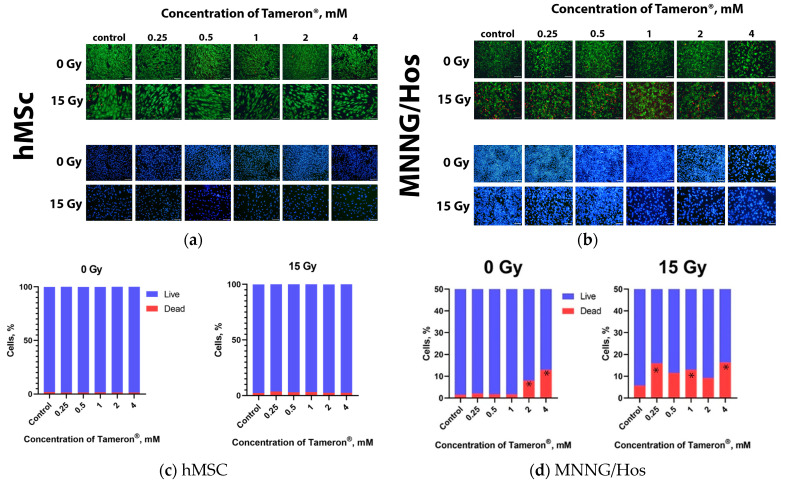
Live/dead culture test. Representative fluorescence images of the LIVE/DEAD analysis of of hMSCs (**a**) and MNNG/Hos osteosarcoma cells (**b**) after pretreatment with various concentrations of Tameron^®^ (0.25–4 mM) and exposure to X-ray radiation (15 Gy). Scale bar—100 µm. Green indicates stained with SYTO9, while red indicates dead cells stained with propidium iodide, blue—nuclei of all cells. Cell viability of of hMSCs (**c**) and MNNG/Hos osteosarcoma cells (**d**) after pretreatment with various concentrations of Tameron^®^ (0.25–4 mM) and exposure to X-ray radiation (15 Gy). * *p* ≤ 0.05 vs. untreated control, and the *p*-value was estimated using one-way ANOVA (*n* = 6).

Next, a quantitative analysis of dead cells (live/dead assay) was carried out, including exposure to high doses of X-rays (15 Gy) (Figure 4). The analysis showed that all tested concentrations of Tameron^®^ did not lead to an increase in the number of dead cells, even at high concentrations of the drug (2 and 4 mM) (Figure 4c). Analysis of the micrographs showed that hMSC cells after irradiation (15 Gy) in the presence of Tameron^®^ increase in their size, a feature typical for cell senescence (Figure 4a). At the same time, the analysis of the nuclear apparatus of cells after irradiation showed a low level of its damage (the presence of micronuclei and chromatin condensation) after irradiation in the presence of all concentrations (0.25–4 mM) of Tameron^®^, which indicates the genoprotective effect of the drug under conditions of exposure to ionizing radiation. Analysis of hMSC cells showed that the number of dead cells after irradiation in the presence of Tameron^®^ did not increase at all tested concentrations (0.25–4 mM), which confirms the radioprotective activity. At the same time, co-incubation of human osteosarcoma cells with Tameron^®^ without irradiation showed a significant increase in the number of dead cells at high concentrations (2 and 4 mM) (Figure 4b). Irradiation of MNNG/Hos cells at a dose of 15 Gy leads to a significant increase in the number of dead cells at all studied concentrations of Tameron^®^, with the maximum death observed at concentrations of 0.25 and 4 mM (Figure 4d). Analysis of the nuclear apparatus of human osteosarcoma cells shows a high level of damage, including chromatin condensation, a large number of micronuclei, and irregularly shaped nuclei.

The choice of hMSCs derived from dental pulp as the normal cell model was motivated by their clinical relevance as a radiosensitive stromal population, their well-characterized phenotype, and the practical advantage of enabling a direct tissue–context comparison with the MNNG/Hos osteosarcoma line. We acknowledge, however, that bone marrow-derived mesenchymal stromal cells and primary hematopoietic progenitors—which are among the most radiosensitive cell populations in vivo and are directly at risk during skeletal radiotherapy—would provide a more immediately translatable model for radioprotection studies. Validation of the observed effects in such cell populations, as well as in additional osteosarcoma lines with varying genetic backgrounds, represents an important direction for future work.

### 2.4. Tameron^®^ Acts as an Antioxidant, Reducing Intracellular ROS and Increasing Glutathione

**Figure 5:**
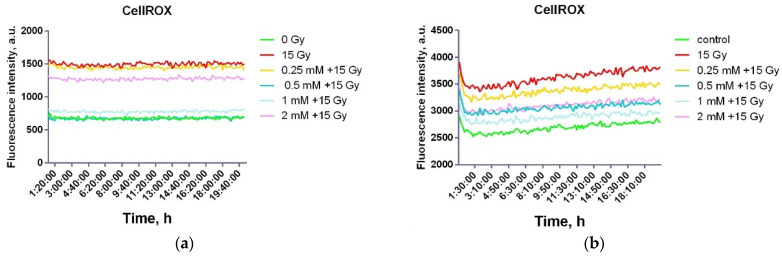

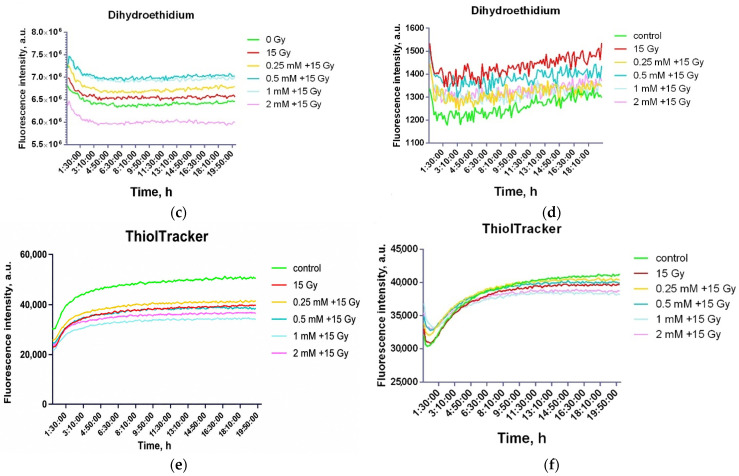
Intracellular ROS level analysis in hMSCs (**a**,**c**,**e**) and MNNG/Hos osteosarcoma cells (**b**,**d**,**f**) after pretreatment with Tameron^®^ (0.25–4 mM) and X-ray exposure (15 Gy). Cells were stained with CellROX (**a**,**b**), DHE (**c**,**d**) and ThiolTracker (**e**,**f**) selective dyes. For each experimental group, eight wells of the plate were analyzed (*n* = 8).

To identify the effect of Tameron^®^ on the metabolism of reactive oxygen species (ROS) under conditions of radiation-induced oxidative stress, a quantitative analysis of the ROS level was carried out using two fluorescent probes: CellROX and dihydroethidium, within 20 h after irradiation using a fluorescent plate reader (Figure 5). The results show that in the case of the CellROX dye, there is a significant increase in the level of fluorescence after irradiation (≈1500 a.u.) (Figure 5a–d). Pretreatment of hMSCs with the drug at a dose of 0.5, 1, and 2 mM led to a significant decrease in the fluorescence level to 750, 800, and 1300 a.u., respectively, which indicates its pronounced antioxidant properties (Figure 5a). A concentration of 0.25 mM under irradiation did not provide a significant decrease in the level of ROS. Cancer cells have shown different dynamics of changes in the intracellular ROS level (Figure 5b). In particular, the baseline level of ROS was increased compared to hMSCs. Irradiation of cancer cells led to a decrease in the fluorescence intensity of the probe in the first hour of observation, but after that, there was a slight increase in the level of ROS. Pretreatment of cells with Tameron^®^ led to a decrease in the level of ROS, which confirms its antioxidant properties in relation to cancer cells. It should be noted that more than 0.5 and 2 mM drug concentrations showed similar dynamics of ROS generation in human osteosarcoma cells. Next, the level of superoxide anion radical was analyzed using a dihydroethidium fluorescent probe in hMSC cells (Figure 5c,d). It was shown that concentrations of 0.5 and 1 mM lead to the preservation of high levels of ROS, while a concentration of 2 mM causes a significant decrease below the control values (relative to non-irradiated control). It should be noted that the situation was different for the osteosarcoma cell culture. All studied concentrations of the drug (0.25–4 mM) also led to a significant decrease in the level of superoxide anion radical and remained at this level for 20 h of detection.

Analysis of the level of non-enzymatic antioxidants obtained using the ThiolTracker selective dye allows us to speak about a different response of hMSC cells and osteosarcoma cells after their irradiation in the presence of Tameron^®^ (Figure 5e,f). This dye binds to reduced glutathione in the cytoplasm and fluoresces at 525 nm. In particular, it was shown that the initial fluorescence intensity of this dye, and hence the level of reduced glutathione in human MSCs, is higher than in MNNG/Hos osteosarcoma cells, which is associated with a high baseline level of ROS, as shown earlier (Figure 5a,b). All concentrations tested had no effect on reduced glutathione levels. Tameron^®^ (0.25 mM) significantly increased the level of reduced glutathione in the first hours, and then kept it at a high level throughout the observation period. For the MNNG/Hos cell culture, in the first hour after irradiation, there is a sharp decrease in the level of reduced glutathione in the presence of various concentrations of Tameron^®^, and after 2 h, there is a significant increase, which may be associated with the efficiency of glutathione reductase.

### 2.5. Tameron^®^ Enhances DNA Repair After Irradiation in the Normal hMSC Cells, While Exhibiting a Radiosensitizing Effect on MNNG/Hos Cells

**Figure 6:**
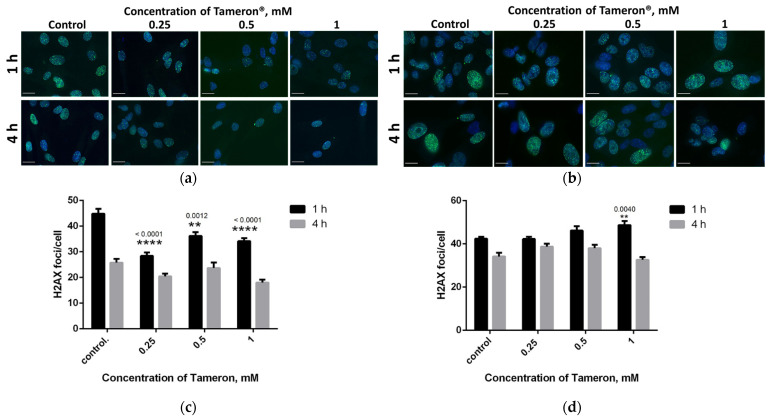
Analysis of the level of DNA double-strand breaks in hMSCs (**a**,**c**) and MNNG/Hos osteosarcoma cells (**b**,**d**) after pretreatment with Tameron^®^ (0.25–4 mM) and X-ray irradiation (1.5 Gy). Cells were stained with FITC-labeled antibodies to phosphorylated histone γH2AX. Scale bars = 20 µm. ** and **** *p*-values were estimated using one-way ANOVA (*n* = 30).

To identify the effect of the drug on the DNA repair system, we analyzed the number of phosphorylated γH2AX loci after X-ray irradiation at a dose of 1.5 Gy (Figure 6). Analysis of the number of DNA double-strand breaks by immunostaining of phosphorylated γH2AX loci after X-ray irradiation at a dose of 1.5 Gy revealed a different response of cell cultures to their preliminary treatment with Tameron^®^ at various concentrations (0.25–1 mM). Pretreatment of human MSCs with Tameron^®^ at all studied concentrations (0.25–1 mM) 1 h after irradiation revealed a significant decrease in the number of DNA double-strand breaks compared to the untreated control (* *p* ≤ 0.0001 for 0.25 mM, *p* ≤ 0.0012 for 0.5 mM, *p* ≤ 0.0001 for 1 mM). At the same time, 4 h after irradiation, only the maximum concentration of Tameron^®^ showed a difference with the control group. Lower concentrations of the drug (0.25–0.5 mM) 4 h after irradiation did not provide a significant acceleration of DNA double-strand break repair. In the case of human osteosarcoma cells of the MNNG/Hos line, the drug not only did not reduce the number of DNA double-strand breaks at concentrations of 0.25 and 0.5 mM, but also significantly increased (* *p* ≤ 0.004) at the maximum concentration (1 mM). Moreover, 4 h after irradiation, the number of DNA double-strand breaks in all studied concentrations did not have significant differences from the control group (without exposure to Tameron^®^). Thus, it can be concluded that Tameron^®^ is able to exert a selective effect on the DNA double-strand break repair system, exhibiting a radiosensitizing effect on the osteosarcoma cells under exposure to ionizing radiation.

### 2.6. Transcriptomic Analysis of the MNNG/Hos Cells After X-Ray Irradiation

To elucidate the molecular mechanisms underlying the differential radiomodulatory effects of Tameron^®^ on MNNG/Hos osteosarcoma cells, we performed nanopore-based transcriptomic analysis on Tameron^®^-treated and untreated MNNG/Hos cells with and without 15 Gy X-ray irradiation. Tameron^®^ was used at two concentrations (0.25 mM and 2 mM). The experimental comparison groups are described in Table 1. Differential gene expression analysis was performed for each group on day 1 and day 3 after treatment. Groups treated with 0.25 mM Tameron^®^ showed no significant differential expression relative to their respective controls, consistent with the sub-effective concentration observed in cellular assays.

#### 2.6.1. Changes in Gene Expression on the First Day After Treatment

On day 1, significant gene expression changes were detected in two pairwise comparisons involving 2 mM Tameron^®^: (i) non-irradiated cells treated with 2 mM Tameron^®^ (group 1) versus untreated controls, and (ii) irradiated cells treated with 2 mM Tameron^®^ (group 3) versus irradiated untreated cells (group 2). The overall number of differentially expressed genes at this early time point was low, consistent with the mild and gradual onset of the drug’s transcriptional effects. Supplementary data for this section are provided in Figures S1 and S4; Tables S1 and S4.

##### Group 1 vs. Control: Effect of 2 mM Tameron^®^ Without Irradiation (Day 1)

**Figure 7:**
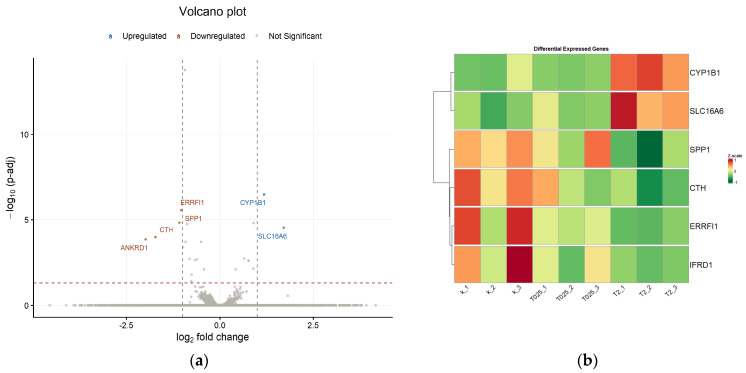
(**a**) Volcano plot of gene expression changes in the 2mM Tameron^®^-treated group without irradiation (group 1) to the corresponding control group (cells without irradiation or any drug treatment) after 1 day. The x-axis (log2FoldChange) is the logarithm of the expression change, and the y-axis (−log10(P-adj)) is the negative decimal logarithm of the adjusted *p*-value. (**b**) Heatmap of the relative gene expression changes between the 2 mM Tameron^®^ -treated group 1 (T2 1-3), 0.25 mM Tameron^®^-treated group 5 (T025 1-3), and the unirradiated, Tameron^®^-untreated control group (k 1-3). The scale reflects the Z-score value—a statistical measure that quantifies the distance between a data point and the mean of a dataset, indicating how many standard deviations a data point is from the mean of the distribution.

Six genes were differentially expressed in non-irradiated cells treated with 2 mM Tameron^®^ compared to untreated controls (Figure 7a,b). Four genes—*ANKRD1*, *CTH*, *SPP1*, and *ERRFI1*—were downregulated, while two—*CYP1B1* and *SLC16A6*—were upregulated. Taken together, these early changes suggest an antiproliferative and antitumorigenic shift in MNNG/Hos cells: suppression of pro-survival and pro-growth signals is reflected by the downregulation of the invasion marker *SPP1*/osteopontin [24,25,26,27,28], the oncogenic *EGFR* modulator *ERRFI1* [29,30,31,32,33], the interferon gamma-associated co-regulator *IFRD1* [34,35,36,37], and the H_2_S-producing cytoprotective enzyme CTH [38,39,40,41,42]. Concurrently, the upregulation of *CYP1B1* may indicate enhanced metabolism of endogenous pro-carcinogenic substrates [43], and *SLC16A6*-mediated taurine transport may reflect a compensatory antioxidant response [44,45]. These transcriptomic shifts are consistent with the cytotoxic effects of Tameron^®^ at higher concentrations observed in Figure 4.

Among the downregulated genes, *ERRFI1* (*MIG6*) encodes a negative regulator of EGFR family signaling that is normally induced during cell growth and stress [29,30,31,32]; its reduced expression may paradoxically reflect the Tameron^®^-mediated suppression of the oncogenic EGFR pathway [33,46]. *SPP1* (osteopontin) is a well-established driver of osteosarcoma proliferation, migration, and invasiveness [28], and its downregulation here is consistent with a potential antimetastatic effect of the drug. *CTH* encodes cystathionine gamma-lyase, which produces the cytoprotective signaling molecule hydrogen sulfide (H_2_S); downregulation of *CTH* reduces H_2_S-dependent NF-κB p65 sulfhydration, thereby lowering the apoptotic threshold in cancer cells [40,41]. Among the upregulated genes, *CYP1B1* is a cytochrome P450 enzyme involved in the metabolism of procarcinogens and endogenous steroids [43], while *SLC16A6* facilitates transmembrane taurine transport and supports mitochondrial function and ER antioxidant defense [44,45].

##### Group 3 vs. Group 2: Effect of 2 mM Tameron^®^ Under Irradiation (Day 1)

**Figure 8:**
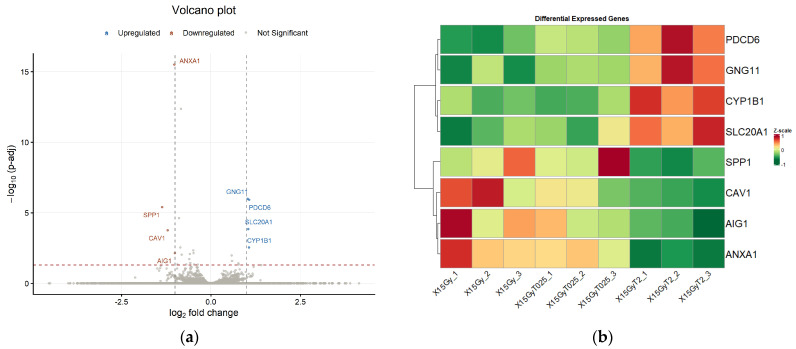
(**a**) Volcano plot of gene expression changes in the 2mM Tameron^®^-pretreated group with irradiation (group 3) compared to the group with irradiation but without drug treatment (group 2) after 1 day. The x-axis (log2FoldChange) is the logarithm of the expression change, and the y-axis (−log10(P-adj)) is the negative decimal logarithm of the adjusted *p*-value. (**b**) Heatmap of the relative gene expression changes between the irradiated, Tameron^®^-untreated group 2 (X15Gy 1-3), irradiated, 2 mM Tameron^®^-treated group 3 (X15GyT2 1-3), and irradiated, 0.25 mM Tameron^®^-treated group 4 (X15GyT025 1-3). The scale reflects the Z-score value—a statistical measure that quantifies the distance between a data point and the mean of a dataset, indicating how many standard deviations a data point is from the mean of the distribution.

Eight genes were differentially expressed in irradiated cells pretreated with 2 mM Tameron^®^ (group 3) compared to irradiated untreated cells (group 2) at day 1 (Figure 8). Four genes—*SPP1*, *CAV1*, *ANXA1*, and *AIG1*—were downregulated; four genes—*PDCD6*, *GNG11*, *SLC20A1*, and *CYP1B1*—were upregulated. The overall pattern indicates that Tameron^®^ potentiates the pro-apoptotic and anti-invasive effects of ionizing radiation in osteosarcoma cells, consistent with the radiosensitizing activity demonstrated in Figure 3 and Figure 4.

Among the downregulated genes, *SPP1* showed more pronounced suppression than in the non-irradiated condition, further supporting the potential antimetastatic effect of Tameron^®^ in combination with radiotherapy [47,48]. *CAV1* (caveolin-1), a scaffold protein linking integrins to the Ras–ERK and Wnt/β-catenin pathways [49,50,51,52,53,54], was also downregulated; while *CAV1* has tumor-suppressive roles in some contexts, its downregulation here may reflect disruption of pro-survival membrane signaling in cancer cells. *ANXA1*, encoding annexin A1, an anti-inflammatory and immune-modulatory protein whose loss is commonly observed in tumors [55,56,57,58,59], was reduced, suggesting impaired cancer cell immune evasion under combined treatment. *AIG1*, an androgen-induced lipid hydrolase and NFAT pathway activator [60,61,62], was also suppressed, consistent with reduced lipid-mediated survival signaling.

Among the upregulated genes, *PDCD6* encodes a calcium-binding pro-apoptotic protein that promotes TNFα-dependent apoptosis via NF-κB activation and upregulation of *Bax*, *p53*, and *p21* [63,64,65]; its upregulation suggests active engagement of apoptotic pathways in Tameron^®^-treated irradiated cells. *SLC20A1*, a sodium–phosphate cotransporter that also activates NF-κB and MAPK signaling [66,67,68], was elevated, consistent with stress signaling activation. *CYP1B1* upregulation continued from the non-irradiated condition. *GNG11*, a G-protein gamma subunit frequently downregulated in cancers and proposed as a prognostic marker [69], was upregulated, which may reflect drug-induced restoration of normal G-protein signaling.

#### 2.6.2. Changes in Gene Expression on the Third Day After Treatment

**Figure 9:**
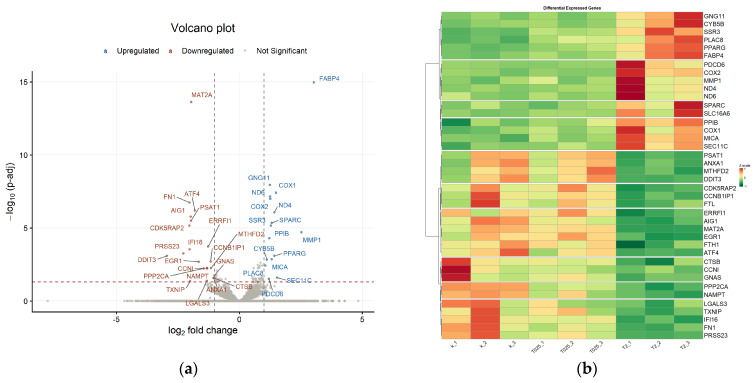
(**a**) Volcano plot of gene expression changes in the 2mM Tameron^®^-treated group without irradiation (group 1) to the corresponding control group (cells without irradiation or any drug treatment) after 3 days. The x-axis (log2FoldChange) is the logarithm of the expression change, and the y-axis (−log10(P-adj)) is the negative decimal logarithm of the adjusted *p*-value. (**b**) Heatmap of the relative gene expression changes between the 2 mM Tameron^®^-treated group 1 (T2 1-3), 0.25 mM Tameron^®^-treated group 5 (T025 1-3), and the unirradiated, Tameron^®^-untreated control group (k 1-3). The scale reflects the Z-score value—a statistical measure that quantifies the distance between a data point and the mean of a dataset, indicating how many standard deviations a data point is from the mean of the distribution.

By day 3, the transcriptomic landscape of Tameron^®^-treated non-irradiated MNNG/Hos cells (group 1) underwent a substantial reorganization, with 40 differentially expressed genes identified compared to only six on day 1 (Figure 9). This temporal progression suggests that the initial, relatively mild transcriptomic perturbation evolves into a broader and more sustained cellular response, likely reflecting delayed drug effects on gene regulatory networks. Additional materials on this section are presented in the Supplementary Materials (Figures S2 and S3; Tables S2 and S3).

#### 2.6.3. Pathway Enrichment Analysis

Gene Ontology (GO)-based pathway enrichment analysis was performed to identify higher-order biological processes associated with Tameron^®^-induced transcriptomic changes [70].

On day 1, non-irradiated Tameron^®^-treated cells (group 1 vs. control) showed decreased enrichment of pathways related to aerobic respiration, mitochondrial electron transport-coupled ATP synthesis, cellular oxygen and hypoxia responses, and NADH dehydrogenase complex activity. No pathways with increased enrichment could be annotated at this time point. This early downregulation of oxidative phosphorylation is consistent with a transient shift toward anaerobic (Warburg-type) metabolism [71], likely reflecting drug-induced metabolic stress in cancer cells.

By day 3, the pathway enrichment profile was markedly reversed. Pathways associated with aerobic respiration, mitochondrial ATP synthesis, oxygen response, NADH dehydrogenase activity, oxidative stress response (including brown adipose tissue-related thermogenic pathways [72]), and general cell proliferation and migration showed increased enrichment. Conversely, pathways related to circadian DNA-templated development, autophagolysosomal processing, ferric iron accumulation (associated with CA3/CA9-mediated oxidative stress protection), and leucine zipper transcription factor activity showed decreased enrichment. Together, the day 1 to day 3 trajectory in non-irradiated cells suggests an initial metabolic stress that is successfully resolved by day 3, with restoration of aerobic respiration and a recovery of cellular activity.

In irradiated Tameron^®^-treated cells (group 3 vs. group 2) at day 1, GO analysis only identified increased enrichment in pathways related to the negative regulation of cell adhesion, epithelial cell migration, and angiogenesis. The activation of pathways associated with cell migration and angiogenesis warrants careful interpretation. These changes likely reflect a transient stress-adaptive response rather than a true pro-tumorigenic program, given the concurrent upregulation of pro-apoptotic markers (PDCD6) and the net increase in cell death observed at the cellular level (Figure 4). Notably, the NRF2-mediated oxidative stress response pathway—a known contributor to intrinsic radioresistance in cancer cells [73]—was not activated in Tameron^®^-treated MNNG/Hos cells, suggesting that the drug does not trigger compensatory antioxidant resistance mechanisms in tumor cells. Nevertheless, these observations underscore the importance of validating the selectivity of Tameron^®^ action in more complex in vivo models before clinical translation.

To assess whether the transcriptomic changes observed in Tameron^®^-pretreated MNNG/Hos cells are consistent with broader radiation and stress response signatures in cancer cell lines, we cross-referenced our differentially expressed genes against publicly available databases. The ASTRA database (Atlas of Stress Response Activity), which catalogs transcriptomic responses to oxidative stress, DNA damage, heat shock, and hypoxia across human cancer cell lines, organizes responses under four stress categories, including H_2_O_2_-induced oxidative stress and UV/radiation-induced DNA damage [74]. Consistent with patterns reported in ASTRA for DNA damage responses in cancer cell lines, Tameron^®^-treated and irradiated MNNG/Hos cells showed an upregulation of pro-apoptotic signaling (PDCD6), whose gene product promotes TNFα-dependent apoptosis through NF-κB pathway activation and upregulation of Bax, p53, and p21 [75], and a downregulation of invasion-associated genes. Notably, SPP1 (osteopontin), which was consistently downregulated in our dataset, is known to be significantly overexpressed in osteosarcoma tumors compared to normal bone tissue across multiple independent cohorts [76], and its downregulation has been associated with reduced tumor progression potential. Similarly, CAV1 downregulation is consistent with its described role in promoting cancer cell survival and radioresistance through modulation of intracellular redox signaling. The NRF2-mediated oxidative stress response pathway, which has been described as a contributor to intrinsic radioresistance in cancer cell lines in large-scale radiogenomic databases [73], was not activated in Tameron^®^-treated MNNG/Hos cells, suggesting that the drug does not trigger compensatory antioxidant resistance mechanisms in tumor cells—a finding with potentially favorable implications for its use as a radiosensitizing agent. Taken together, the transcriptomic profile of Tameron^®^-treated irradiated MNNG/Hos cells aligns with established radiation response signatures characteristic of cells undergoing impaired survival, rather than adaptive radioresistance.

## 3. Materials and Methods

### 3.1. Object of Research

In this paper, we investigated the drug Tameron^®^. The drug Tameron^®^ is sodium aminodihydrophthalazinedione (or otherwise sodium luminol), synthesized using an original technique. We used a sterile packaged lyophilizate of this drug, which was diluted with a saline buffer or cell culture medium to the required concentrations, which were then added to the cell cultures (the general scheme of the experiments is shown in Table S5).

### 3.2. Cell Cultures

The experiments were conducted on a culture of normal (human mesenchymal stem cells isolated from dental pulp—hMSC) and cancer cells (human osteosarcoma of the MNNG/Hos line). The hMSCs were isolated from a third molar bud extracted for orthodontic reasons from a healthy 16-year-old patient. The third molar bud was removed according to the orthodontic indications of the dental clinic "Dr. MUN" (Moscow, Russia) in accordance with the ethics committee after consent was signed by the patient’s parents. Cells were extracted in DMEM/F12 medium (PanEco, Moscow, Russia) containing 200 U/mL penicillin and 200 mg/mL streptomycin (Life Technologies, Carlsbad, CA, USA) with a syringe inserted into the tooth apex, followed by treatment with 0.25% trypsin + 0. 02% EDTA (Life Technologies) for 30 min at 37 °C. The isolated cells were centrifuged for 2 min at 1500 rpm and resuspended to single cells in a culture medium consisting of DMEM/F12 (1:1; Life Technologies) supplemented with 10% fetal bovine serum (FBS). The resulting solution was transferred into 25 mL vials and cultured in 5% CO_2_ at 37 °C with the addition of 10% FBS (HyClone, Marlborough, MA, USA), 100 U/mL penicillin/streptomycin, and 2 mM L-glutamine in DMEM (PanEco). Upon reaching the state of subconfluent cells, the cultured cells were treated with 0.25% EDTA/trypsin solution and added to 75 cm^2^ flasks in a ratio of 1:3. Cells were cultured in DMEM/F12 medium (PanEco,) supplemented with 10% FBS, 100 U/mL penicillin/streptomycin, and 2 mM L-glutamine. In our study, cell cultures of 3–4 passages were used. The MNNG/Hos human osteosarcoma cell culture was obtained from the European Collection of Authenticated Cell Cultures (ECACC) and deposited in the cryobank of the Cell and Tissue Growth Laboratory of the ITEB RAS and cultivated according to a scheme similar to human MSCs.

### 3.3. Antioxidant Effects in Buffer Solutions

A highly sensitive method of enhanced chemiluminescence in the “luminol-4-iodophenol-horseradish peroxidase” system was used, which allows quantitative determination of the concentration of hydrogen peroxide in aqueous solutions. Chemiluminescence intensity was measured on a Beta-1 liquid scintillation counter (Medapparatura, Kyiv, Ukraine) in single photon counting mode (without a coincidence scheme). The sensitivity of the method makes it possible to determine H_2_O_2_ at a concentration of 0.1 nM. After irradiation, solution samples (3 mL) were placed in polypropylene vials (Beckman, Brea, CA, USA) and 150 µL of a freshly prepared “counting solution” containing: Tris-HCl buffer pH 8.5, 4-iodophenol, luminol in a ratio of 10:3:1 were added, as well as 0.3–0.7 µL of concentrated horseradish peroxidase solution. The concentration of H_2_O_2_ formed was determined using a calibration curve. It was obtained by measuring samples containing H_2_O_2_ of known concentration. The concentration of the stock solution of hydrogen peroxide used for calibration was determined spectrophotometrically at λ = 240 nm using a known molar absorption coefficient of 43.6 M^−1^ × cm^−1^ on a Cary 100 Scan spectrophotometer (Agilent Technologies, Santa Clara, CA, USA) in the UV-visible region spectrum. The following reagents were used in the work: hydrogen peroxide, tris(hydroxymethyl)aminomethane, horseradish peroxidase, 4-iodophenol (Sigma-Aldrich, St. Louis, MO, USA), luminol (AppliChem, Darmstadt, Germany), and hydrochloric acid (HCl) (Reakhim, Moscow, Russia). Bidistilled H_2_O pH 5.6 with a specific electrical conductivity of 200 µS/m, saturated with atmosphere for 24 h, was used in the experiments. The concentration of dissolved O_2_ in H_2_O was about 270 µM. All reagents were used without further purification.

### 3.4. Irradiation of Cell Cultures

Cells were irradiated using a RUT-15 therapeutic X-ray machine (Mosrentgen, Moscow, Russia) at a dose of 15 Gy at a dose rate of 1 Gy/min, a voltage of 200 kV, a focal length of 37.5 cm, and a current of 20 mA. Cells were irradiated in 96- and 6-well culture plates or culture flasks (12.5 cm^2^).

### 3.5. Intracellular ROS Detection

The level of intracellular ROS after irradiation was determined using 2 selective fluorescent dyes: CellROX (Invitrogen,) and dihydroethidium (Sigma Aldrich). The CellROX dye (2 µM) is able to effectively penetrate into the cell and, when oxidized with reactive oxygen species (mainly peroxides), to exhibit bright green photostable fluorescence. Upon subsequent binding to DNA, it has absorption/emission maxima at ~485/520 nm. Dihydroethidium (4 µM) was used to analyze the level of hydroxyl radical after irradiation in the presence of Tameron^®^. Cells were seeded in 96-well culture plates at a density of 20 thousand cells/cm^2^. The seeding density of MNNG/Hos cells was 15000 cells/cm^2^. After cultivation for 14 h in DMEM/F12 medium (1:1) with 10% addition of FBS at a temperature of 37 °C, 95% humidity in an atmosphere with 5% CO_2_, various concentrations of Tameron^®^ were added. After 3 h of incubation, cells were stained with CellRox and dihydroethidium fluorescent probes. 30 min after staining, the culture medium was replaced with a medium without phenol red, and the cells were irradiated with X-rays at a dose of 15 Gy. The intensity of fluorescence was recorded with a probe on a tablet reader, H1 Synergy Biotek (Winuskey, VT, USA), for 20 h after irradiation.

### 3.6. Analysis of the Level of Non-Enzymatic Antioxidants

Mesenchymal stem cells were seeded in 96-well culture plates at a density of 2 × 10^4^ cells/cm^2^. The seeding density of MNNG/Hos cells was 1.5 × 10^4^ cells/cm^2^. After cultivation for 14 h in DMEM/F12 medium (1:1) with 10% addition of FBS at a temperature of 37 °C, 95% humidity in an atmosphere with 5% CO_2_, various concentrations of Tameron^®^ were introduced. After 3 h of incubation, cells were stained with ThiolTracker (estimating the cellular level of reduced glutathione). After staining for 30 min, the culture medium without phenol red was added, and the cells were irradiated with X-rays at a dose of 15 Gy. Fluorescence was recorded on a tablet reader, H1 Synergy Biotek, for 20 h.

### 3.7. MTT Assay

The metabolic activity of cell cultures was determined by the MTT test, the principle of which is based on the reduction of a colorless water-soluble tetrazolium salt (3-[4,5-dimethylthiazol-2-yl]-2,5-diphenyltetrazolium bromide) to insoluble formazan crystals by cellular dehydrogenases. Cells were seeded in 96-well plates and cultured in an atmosphere containing 5% CO_2_ at 37 °C. Six hours after inoculation, the medium was replaced with a medium containing various concentrations of sodium luminol (from 0.1 to 2 mM). Viability was determined 72 h after adding the drug.

To determine hMSC cell numbers, a calibration curve was first constructed by seeding cells at densities of 0.5, 1.0, 5.0, 10.0, and 20.0 × 10^3^ cells/well. Cells were seeded in 96-well plates and cultured in a 5% CO_2_ atmosphere at 37 °C. After 24 h, an MTT assay was performed to obtain a cell number vs. optical density calibration curve. To investigate the effect of Tameron^®^ on the proliferative activity of hMSCs under irradiation, cells were seeded in 96-well plates and cultured for 16 h (overnight). Next, the cells were treated with sodium luminol at various concentrations (0.25–2 mM) and irradiated at a dose of 5 Gy. Following irradiation, hMSCs were cultured for 8 days, after which an MTT assay was performed. Cell numbers were determined using the calibration curve.

### 3.8. Live/Dead Assay

The viability of cells cultured in the presence of sodium luminol was assessed using a Carl Zeiss Axiovert 200 microscope. L-7007 LIVE/DEAD BacLight Bacterial Viability Kit (Invitrogen) was used for analysis, which includes SYTO 9 fluorescent dye for staining all cells (absorption—420 nm, emission—580 nm),the dye propidium iodide (PI), which stains the nuclei of only dead cells (absorption 488 nm and emission 640 nm). Hoechst 33258, which stains the nuclei of all cells (absorption 405 nm and emission 430–480 nm) A mixture of dyes (1 µg/mL) was added to the medium, and then the culture plate was placed in a CO_2_ incubator for 15 min. Micrographs were taken after washing the cells with phosphate-buffered saline.

### 3.9. Clonogenic Assay

Cells were seeded in 12.5 cm^2^ flasks and cultured for 16 h (overnight). Next, the cells were treated with sodium luminol at various concentrations (0.25–2 mM) and irradiated at a dose of 5 Gy. Immediately after irradiation, the cells were seeded in 6-well plates at a concentration of 2000 cells per well in the culture medium DMEM/F12 + 10% FBS and cultured for 8 days. Then the cells were washed three times with phosphate-buffered saline and fixed in 4% paraformaldehyde solution and stained with 0.1% crystal violet. Cell aggregations of more than 50 cells were considered 1 colony.

### 3.10. DNA Double-Strand Break Analysis

Cells were seeded at a density of 2 × 10^4^ cm^2^, cultured for 16 h (overnight), and sodium luminol was added at various concentrations (0.25, 0.5, and 1 mM). Next, the cells were cultured in the presence of Tameron^®^ for 24 h and exposed to X-ray irradiation at a dose of 1.5 Gy. Analysis of the number of DNA double-strand breaks was performed 1 h and 4 h after irradiation. After irradiation, the cells were washed twice with phosphate-buffered saline (PBS, pH 7.4). Cells were then fixed with 4% paraformaldehyde in PBS for 10 min, permeabilized with 0.3% Triton X-100 in PBS for 5 min, washed thoroughly with PBS, and blocked in 1% BSA in PBS for 30 min at room temperature. Primary antibodies, monoclonal mouse γH2AX (ab195188 Recombinant, Anti-gamma γH2AX (phospho S139), (Alexa Fluor^®^ 488), Abcam, Waltham, Boston, MA, USA) were used for immunostaining. DSB repair foci were imaged using Zeiss Axiovert Observer 200M inverted microscopy (Carl Zeiss Microscopy, Jena, Germany) using a 63× objective (NA, 0.7). Analysis of cells stained with γH2AX was performed using the FindFoci ImageJ (v. 1.5, National Institutes of Health [NIH], Bethesda, MD, USA) plug-in for automatic recognition of lesions [77]. For each experimental group, at least 20 visual fields were analyzed.

### 3.11. Expression Level Analysis by Nanopore Sequencing

#### 3.11.1. mRNA Isolation

Cells after incubation with various concentrations of Tameron^®^ were collected and stored in EverFresh RNA solution (Sileks, Moscow, Russia). mRNA was isolated from cells using magnetic particles (a kit from Sileks, Russia). For this, the cell suspension was centrifuged at 12,000× *g* for 15 min. mRNA was eluted in 10 µL of RNAse-free water. Quantification of mRNA was obtained on the Qubit RNA HS assay (Thermofisher, Waltham, MA, USA).

#### 3.11.2. Obtaining cDNA, PCR—Amplification of the cDNA Library

cDNA was obtained from 400 ng of mRNA using the reverse transcription method with the Mint SK001 kit (Evrogen, Moscow, Russia) according to the manufacturer’s recommendations. The synthesis reaction was carried out for 1.5 h at a temperature of 42 °C. Next, the resulting cDNA was amplified using universal primers, and the PCR reaction components included in the Mint cDNA kit. Initially, analytical amplification was performed to determine the optimal number of PCR cycles. After that, a PCR reaction was carried out in 50 μL, using 2 μL of the cDNA product with the optimal number of amplification cycles. At the end of the reaction, 1 μL Exonuclease I (NEB M0293, Ipswich, MA, USA) was added directly to each PCR tube, and the reactions were incubated for 15 min at 37 °C, and heat-inactivated at 80 °C for 15 min. To control the results of the reaction, the amplification products were visualized using horizontal agarose gel electrophoresis.

#### 3.11.3. Preparation of cDNA Library for Sequencing on the MinION Platform

Previously, the cDNA library of each analyzed sample was purified using AMPure XP beads (Beckman Coulter, A63880, Brea, CA, USA). To do this, 51 µL of the reaction mixture with amplicons was transferred into a 0.5 mL Eppendorf DNA LoBind tube, and 41 µL of particles were added, pipetted, and incubated in a Hula Mixer for 5 min. The samples were then spun down briefly and placed on a magnet to pellet the AMPure XP beads. The supernatant was carefully discarded, and the pellet was washed 2× with 500 µL of fresh 70% EtOH. After drying (30 s), the pellet was resuspended in 21 μL of water and incubated at RT for 2 min. The samples were placed on a magnet, and the supernatant was saved. In 1 μL of the resulting purified cDNA library, the concentration was measured using the QuDye HS kit (Lumiprope, Moscow, Russia, Cat 12102). The cDNA library was prepared using NBD-104 and NBD-114 native barcoding kits (Oxford Nanopore, Oxford, UK). Then, 18 indexed samples were loaded into the Min106D cell for sequencing. Indexing was carried out according to the original protocol. Initially, the stage of DNA repair and end-prep was carried out. To do this, pre-mixed 0.75 µL of Ultra II End-prep enzyme mix from the NEBNext Ultra II End repair/dA-tailing Module kit (NEB, 7546) and 1.25 µL Ultra II End-prep reaction buffer and incubated at 20 C for 5 min, 80 C for 5 min. The resulting mixture was then purified with 15 µL AMPure XP beads and eluted in 10 µL mQ water. For the next step, native index ligation, 3.75 µL of the purified library, 1.25 µL of the index from the kit, and 5 µL of Blunt/TA Ligase Master Mix (NEB, M0367) were used. The resulting mixture was incubated for 20 min at 20 °C, then 1 µL of 0.5 M EDTA was added, and all indexed samples were mixed in a 0.5 mL Eppendorf DNA LoBind tube. Next, 80 µL of AMPure XP beads were added, pipetted, and incubated in a Hula Mixer for 10 min. The samples were then spun down briefly and placed on a magnet to pellet the AMPure XP beads. The supernatant was carefully discarded, and the pellet was washed 2× with 700 µL of fresh 70% EtOH. After drying (30 s), the pellet was resuspended in 35 μL of water and incubated at 37 C for 10 min. The samples were placed on a magnet, and the supernatant was saved. In 1 μL of the resulting purified cDNA library, the concentration was measured using the QuDye HS kit (Lumiprope, Russia, Cat 12102). Sequencing of the prepared cDNA libraries was performed using a MinION sequencer (Oxford Nanopore, Oxford, UK) on a FLO-MIN106D cell (Oxford Nanopore, Oxford, UK) flow cell using the MinKNOW basic software (v. 24.06.8, Oxford Nanopore, UK).

#### 3.11.4. Bioinformatic Analysis of Nanopore Transcriptomic Data

Primary analysis of the obtained data (basecalling and demultiplexing) was performed using the Guppy v6.2.1 program (using basecall model sup c the minimum q-score a read must attain to pass qscore filtering 10). For primary bioinformatic data processing (mapping FASTQ data and calculating gene expressions), the pipeline-transcriptome-de (https://github.com/nanoporetech/pipeline-transcriptome-de, accessed on 29 May 2022) was used. Gene expression heatmaps, principal component analysis (PCA), k-means clustering of DEGs, the analysis of co-expression patterns, the enriched analysis based on the Gene Ontology (GO) database, and pathway analysis using the Parametric Gene Set Enrichment Analysis (PGSEA) package (v. 1.30.0) in the studied cell samples were explored using iDEP.91 (edgeR package, https://bioinformatics.sdstate.edu/idep90/, accessed on 8 October 2025) [78]. For differential gene expression, multiple-testing correction was applied using the Benjamini–Hochberg procedure.

### 3.12. Statistical Processing

Statistical analysis was carried out using the GraphPad Prism 8.0 software. All experimental data were presented as mean ± standard deviation (SD) and tested for statistically significant differences using one-way analysis of variance (ANOVA). Differences between experimental groups were considered statistically significant at a *p*-value ≤ 0.05.

## 4. Conclusions

The results obtained confirm that Tameron^®^ acts as an antioxidant and a radiomodulatory agent, exhibiting different molecular mechanisms of action on normal and cancer cells. In aqueous solutions, Tameron^®^ demonstrated antioxidant properties, significantly reducing the hydrogen peroxide formed after X-ray irradiation. In vitro, it also demonstrates pronounced antioxidant properties, effectively reducing the level of intracellular ROS and increasing the concentration of glutathione in both normal and tumor cells, although with slightly different mechanisms of action. Tameron^®^ shows pronounced radioprotective properties on normal hMSCs, increasing their viability and clonogenic activity after irradiation; however, in the case of cancer cells, this effect is not manifested. Moreover, Tameron^®^ demonstrates a radiosensitizing effect, causing damage and leading to cell death in cancer MNNG/Hos cells. It also enhances the repair of double-stranded DNA breaks in normal hMSCs after irradiation, inhibiting this process in osteosarcoma cancer cells. Transcriptomic analysis revealed that Tameron^®^ significantly modulates key genes and signaling pathways responsible for antioxidant activity, cell proliferation, migration, invasiveness, and angiogenesis in both irradiated and non-irradiated MNNG cancer cells. In general, our results indicate that Tameron^®^ can be considered as a promising redox-active drug for the supportive treatment of cancer patients receiving radiation therapy. Meanwhile, it is worth noting that the data obtained in vitro require confirmation in vivo experimental models with intraperitoneal or intravenous administration of sodium aminodihydrophthalazinedione both before and after irradiation, which will allow us to fully talk about the prospects of this drug as a radioprotective/radiomitigatory substance.

## Figures and Tables

**Table 1:** Comparison groups for transcriptomic analysis of the MNNG/Hos cell line.

Group	0.25 mM Tameron^®^	2mM Tameron^®^	15 Gy
Control	−	−	−
Group1	−	+	−
Group2	−	−	+
Group3	−	+	+
Group4	+	−	+
Group5	+	−	−

## Data Availability

The original contributions presented in this study are included in the article/Supplementary Materials. Further inquiries can be directed to the corresponding author.

## Supplementary Materials

The following supporting information can be downloaded at https://www.mdpi.com/article/10.3390/ijms27125272/s1.

## References

[1] Siemann D.W., Mendenhall W.M. (2001). The role of amifostine as a radioprotector. Oncology.

[2] Kouvaris J.R., Kouloulias V.E., Vlahos L.J. (2007). Amifostine: The first selective-target and broad-spectrum radioprotector. Oncologist.

[3] King M., Joseph S., Albert A., Thomas T.V., Nittala M.R., Woods W.C., Vijayakumar S., Packianathan S. (2020). Use of Amifostine for Cytoprotection during Radiation Therapy: A Review. Oncology.

[4] Shirazi A., Mihandoost E., Mahdavi S.R., Mohseni M. (2012). Radio-protective role of antioxidant agents. Oncol. Rev..

[5] Xu P., Zhang W.B., Cai X.H., Qiu P.Y., Hao M.H., Lu D.D. (2017). Activating AKT to inhibit JNK by troxerutin antagonizes radiation-induced PTEN activation. Eur. J. Pharmacol..

[6] Ozgen S., Dökmeci D., Akpolat M., Karadağ C.H., Gündüz O., Erbaş H., Benian O., Uzal C., Turan F.N. (2012). The Protective Effect of Curcumin on Ionizing Radiation-induced Cataractogenesis in Rats. Balk. Med. J..

[7] Khan S., Kumar A., Adhikari J.S., Rizvi M.A., Chaudhury N.K. (2015). Protective effect of sesamol against ^60^Co γ-ray-induced hematopoietic and gastrointestinal injury in C57BL/6 male mice. Free Radic. Res..

[8] Sredni B., Albeck M., Kazimirsky G., Shalit F. (1992). The immunomodulator AS101 administered orally as a chemoprotective and radioprotective agent. Int. J. Immunopharmacol..

[9] Shepet’ko E.N., Bychkova N.G., Garmash D.A., Kurbanov A.K. (2011). Efficacy of the immunomodulating therapy in complex treatment of gastric cancer complicated by acute hemorrhage using radical operations. Klin. Khirurhiia.

[10] Lungu G., Kuang X., Stoica G., Wong P.K. (2010). Monosodium luminol upregulates the expression of Bcl-2 and VEGF in retrovirus-infected mice through downregulation of corresponding miRNAs. Acta Virol..

[11] Reddy P.V., Lungu G., Kuang X., Stoica G., Wong P.K. (2010). Neuroprotective effects of the drug GVT (monosodium luminol) are mediated by the stabilization of Nrf2 in astrocytes. Neurochem. Int..

[12] Schumann S., Kaiser A., Nicoletti F. (2020). Immune-Modulating Drug MP1032 with SARS-CoV-2 Antiviral Activity In Vitro: A potential Multi-Target Approach for Prevention and Early Intervention Treatment of COVID-19. Int. J. Mol. Sci..

[13] Scofield V.L., Yan M., Kuang X., Kim S.J., Crunk D., Wong P.K. (2009). The drug monosodium luminol (GVT) preserves thymic epithelial cell cytoarchitecture and allows thymocyte survival in mice infected with the T cell-tropic, cytopathic retrovirus ts1. Immunol. Lett..

[14] Shetty A.K., Attaluri S., Kodali M., Shuai B., Shetty G.A., Upadhya D., Hattiangady B., Madhu L.N., Upadhya R., Bates A. (2020). Monosodium luminol reinstates redox homeostasis, improves cognition, mood and neurogenesis, and alleviates neuro- and systemic inflammation in a model of Gulf War Illness. Redox Biol..

[15] Le Caër S. (2011). Water Radiolysis: Influence of Oxide Surfaces on H2 Production under Ionizing Radiation. Water.

[16] Kuwahara Y., Tomita K. (2020). The Effects of Hydrogen Peroxide and/or Radiation on the Survival of Clinically Relevant Radioresistant Cells. Technol. Cancer Res. Treat..

[17] Berndt T., Richters S., Jokinen T., Hyttinen N., Kurtén T., Otkjær R.V., Kjaergaard H.G., Stratmann F., Herrmann H., Sipilä M. (2016). Hydroxyl radical-induced formation of highly oxidized organic compounds. Nat. Commun..

[18] Andrés C.M.C., Pérez de la Lastra J.M., Juan C.A., Plou F.J., Pérez-Lebeña E. (2022). Chemistry of Hydrogen Peroxide Formation and Elimination in Mammalian Cells, and Its Role in Various Pathologies. Stresses.

[19] Buch K., Peters T., Nawroth T., Sänger M., Schmidberger H., Langguth P. (2012). Determination of cell survival after irradiation via clonogenic assay versus multiple MTT Assay—A comparative study. Radiat. Oncol..

[20] Munshi A., Hobbs M., Meyn R.E. (2005). Clonogenic cell survival assay. Chemosensitivity.

[21] Rai Y., Pathak R., Kumari N., Sah D.K., Pandey S., Kalra N., Bhatt A.N. (2018). Mitochondrial biogenesis and metabolic hyperactivation limits the application of MTT assay in the estimation of radiation induced growth inhibition. Sci. Rep..

[22] Li M., You L., Xue J., Lu Y. (2018). Ionizing Radiation-Induced Cellular Senescence in Normal, Non-transformed Cells and the Involved DNA Damage Response: A Mini Review. Front. Pharmacol..

[23] Saito Y., Nishio K., Akazawa Y.O., Kimura Y., Nakamura M., Yamaguchi H., Yoshida Y., Noguchi N. (2013). Prolonged expression of senescence markers in mice exposed to gamma-irradiation. PLoS ONE.

[24] Merry K., Dodds R., Littlewood A., Gowen M. (1993). Expression of osteopontin mRNA by osteoclasts and osteoblasts in modelling adult human bone. J. Cell Sci..

[25] Choi S.T., Kim J.H., Kang E.J., Lee S.W., Park M.C., Park Y.B., Lee S.K. (2008). Osteopontin might be involved in bone remodelling rather than in inflammation in ankylosing spondylitis. Rheumatology.

[26] Stier S., Ko Y., Forkert R., Lutz C., Neuhaus T., Grünewald E., Cheng T., Dombkowski D., Calvi L.M., Rittling S.R. (2005). Osteopontin is a hematopoietic stem cell niche component that negatively regulates stem cell pool size. J. Exp. Med..

[27] Wang K.X., Denhardt D.T. (2008). Osteopontin: Role in immune regulation and stress responses. Cytokine Growth Factor Rev..

[28] Han X., Wang W., He J., Jiang L., Li X. (2019). Osteopontin as a biomarker for osteosarcoma therapy and prognosis. Oncol. Lett..

[29] Wick M., Bürger C., Funk M., Müller R. (1995). Identification of a Novel Mitogen-Inducible Gene (mig-6): Regulation during G1 Progression and Differentiation. Exp. Cell Res..

[30] Xu D., Makkinje A., Kyriakis J.M. (2005). Gene 33 is an endogenous inhibitor of epidermal growth factor (EGF) receptor signaling and mediates dexamethasone-induced suppression of EGF function. J. Biol. Chem..

[31] Makkinje A., Quinn D.A., Chen A., Cadilla C.L., Force T., Bonventre J.V., Kyriakis J.M. (2000). Gene 33/Mig-6, a transcriptionally inducible adapter protein that binds GTP-Cdc42 and activates SAPK/JNK. A potential marker transcript for chronic pathologic conditions, such as diabetic nephropathy. Possible role in the response to persistent stress. J. Biol. Chem..

[32] Fiorentino L., Pertica C., Fiorini M., Talora C., Crescenzi M., Castellani L., Alemà S., Benedetti P., Segatto O. (2000). Inhibition of ErbB-2 mitogenic and transforming activity by RALT, a mitogen-induced signal transducer which binds to the ErbB-2 kinase domain. Mol. Cell. Biol..

[33] Anastasi S., Lamberti D., Alemà S., Segatto O. (2016). Regulation of the ErbB network by the MIG6 feedback loop in physiology, tumor suppression and responses to oncogene-targeted therapeutics. Semin. Cell Dev. Biol..

[34] Jorgovanovic D., Song M., Wang L., Zhang Y. (2020). Roles of IFN-γ in tumor progression and regression: A review. Biomark. Res..

[35] Vietor I., Huber L.A. (2007). Role of TIS7 family of transcriptional regulators in differentiation and regeneration. Differ. Res. Biol. Divers..

[36] Micheli L., Leonardi L., Conti F., Maresca G., Colazingari S., Mattei E., Lira S.A., Farioli-Vecchioli S., Caruso M., Tirone F. (2011). PC4/Tis7/IFRD1 stimulates skeletal muscle regeneration and is involved in myoblast differentiation as a regulator of MyoD and NF-kappaB. J. Biol. Chem..

[37] Tirone F., Shooter E.M. (1989). Early gene regulation by nerve growth factor in PC12 cells: Induction of an interferon-related gene. Proc. Natl. Acad. Sci. USA.

[38] Steegborn C., Clausen T., Sondermann P., Jacob U., Worbs M., Marinkovic S., Huber R., Wahl M.C. (1999). Kinetics and inhibition of recombinant human cystathionine gamma-lyase. Toward the rational control of transsulfuration. J. Biol. Chem..

[39] Kasamatsu S., Nishimura A., Morita M., Matsunaga T., Abdul Hamid H., Akaike T. (2016). Redox Signaling Regulated by Cysteine Persulfide and Protein Polysulfidation. Molecules.

[40] Kimura H. (2015). Physiological Roles of Hydrogen Sulfide and Polysulfides. Chemistry, Biochemistry and Pharmacology of Hydrogen Sulfide.

[41] Sen N., Paul B.D., Gadalla M.M., Mustafa A.K., Sen T., Xu R., Kim S., Snyder S.H. (2012). Hydrogen sulfide-linked sulfhydration of NF-κB mediates its antiapoptotic actions. Mol. Cell.

[42] Ju Y., Fu M., Stokes E., Wu L., Yang G. (2017). H2S-Mediated Protein S-Sulfhydration: A Prediction for Its Formation and Regulation. Molecules.

[43] Tang Y.M., Wo Y.Y., Stewart J., Hawkins A.L., Griffin C.A., Sutter T.R., Greenlee W.F. (1996). Isolation and characterization of the human cytochrome P450 CYP1B1 gene. J. Biol. Chem..

[44] Jong C.J., Sandal P., Schaffer S.W. (2021). The Role of Taurine in Mitochondria Health: More Than Just an Antioxidant. Molecules.

[45] Pan C., Giraldo G.S., Prentice H., Wu J.-Y. (2010). Taurine protection of PC12 cells against endoplasmic reticulum stress induced by oxidative stress. J. Biomed. Sci..

[46] Uribe M.L., Marrocco I., Yarden Y. (2021). EGFR in Cancer: Signaling Mechanisms, Drugs, and Acquired Resistance. Cancers.

[47] Liu S.J., Zhang D.Q., Sui X.M., Zhang L., Cai Z.W., Sun L.Q., Liu Y.J., Xue Y., Hu G.F. (2008). The inhibition of in vivo tumorigenesis of osteosarcoma (OS)-732 cells by antisense human osteopontin RNA. Cell. Mol. Biol. Lett..

[48] Berge G., Pettersen S., Grotterød I., Bettum I.J., Boye K., Mælandsmo G.M. (2011). Osteopontin—an important downstream effector of S100A4-mediated invasion and metastasis. Int. J. Cancer.

[49] Dupree P., Parton R.G., Raposo G., Kurzchalia T.V., Simons K. (1993). Caveolae and sorting in the trans-Golgi network of epithelial cells. EMBO J..

[50] Kortum R.L., Fernandez M.R., Costanzo-Garvey D.L., Johnson H.J., Fisher K.W., Volle D.J., Lewis R.E. (2014). Caveolin-1 is required for kinase suppressor of Ras 1 (KSR1)-mediated extracellular signal-regulated kinase 1/2 activation, H-RasV12-induced senescence, and transformation. Mol. Cell. Biol..

[51] Xu L., Wang L., Wen Z., Wu L., Jiang Y., Yang L., Xiao L., Xie Y., Ma M., Zhu W. (2016). Caveolin-1 is a checkpoint regulator in hypoxia-induced astrocyte apoptosis via Ras/Raf/ERK pathway. Am. J. Physiol. Cell Physiol..

[52] Galbiati F., Volonte D., Brown A.M., Weinstein D.E., Ben-Ze’ev A., Pestell R.G., Lisanti M.P. (2000). Caveolin-1 expression inhibits Wnt/beta-catenin/Lef-1 signaling by recruiting beta-catenin to caveolae membrane domains. J. Biol. Chem..

[53] Engelman J.A., Wykoff C.C., Yasuhara S., Song K.S., Okamoto T., Lisanti M.P. (1997). Recombinant expression of caveolin-1 in oncogenically transformed cells abrogates anchorage-independent growth. J. Biol. Chem..

[54] Galbiati F., Volonte D., Engelman J.A., Watanabe G., Burk R., Pestell R.G., Lisanti M.P. (1998). Targeted downregulation of caveolin-1 is sufficient to drive cell transformation and hyperactivate the p42/44 MAP kinase cascade. EMBO J..

[55] Buckingham J.C., Flower R.J., Fink G. (2017). Chapter 25—Annexin A1. Stress: Neuroendocrinology and Neurobiology.

[56] Sugimoto M.A., Vago J.P., Teixeira M.M., Sousa L.P. (2016). Annexin A1 and the Resolution of Inflammation: Modulation of Neutrophil Recruitment, Apoptosis, and Clearance. J. Immunol. Res..

[57] Araújo T.G., Mota S.T.S., Ferreira H.S.V., Ribeiro M.A., Goulart L.R. (2021). Annexin A1 as a Regulator of Immune Response in Cancer. Cells.

[58] Patel D.M., Ahmad S.F., Weiss D.G., Gerke V., Kuznetsov S.A. (2011). Annexin A1 is a new functional linker between actin filaments and phagosomes during phagocytosis. J. Cell Sci..

[59] Kaufman M., Leto T., Levy R. (1996). Translocation of annexin I to plasma membranes and phagosomes in human neutrophils upon stimulation with opsonized zymosan: Possible role in phagosome function. Biochem. J..

[60] Erikci Ertunc M., Kok B.P., Parsons W.H., Wang J.G., Tan D., Donaldson C.J., Pinto A.F.M., Vaughan J.M., Ngo N., Lum K.M. (2020). AIG1 and ADTRP are endogenous hydrolases of fatty acid esters of hydroxy fatty acids (FAHFAs) in mice. J. Biol. Chem..

[61] Parsons W.H., Kolar M.J., Kamat S.S., Cognetta A.B., Hulce J.J., Saez E., Kahn B.B., Saghatelian A., Cravatt B.F. (2016). AIG1 and ADTRP are atypical integral membrane hydrolases that degrade bioactive FAHFAs. Nat. Chem. Biol..

[62] Wu G., Sun M., Zhang W., Huo K. (2011). AIG1 is a novel Pirh2-interacting protein that activates the NFAT signaling pathway. Front. Biosci. Elite Ed..

[63] Jung Y.S., Kim K.S., Kim K.D., Lim J.S., Kim J.W., Kim E. (2001). Apoptosis-linked gene 2 binds to the death domain of Fas and dissociates from Fas during Fas-mediated apoptosis in Jurkat cells. Biochem. Biophys. Res. Commun..

[64] Zhu Y., Li Q. (2024). Multifaceted roles of PDCD6 both within and outside the cell. J. Cell. Physiol..

[65] Rho S.B., Song Y.J., Lim M.C., Lee S.H., Kim B.R., Park S.Y. (2012). Programmed cell death 6 (PDCD6) inhibits angiogenesis through PI3K/mTOR/p70S6K pathway by interacting of VEGFR-2. Cell. Signal..

[66] Olah Z., Lehel C., Anderson W.B., Eiden M.V., Wilson C.A. (1994). The cellular receptor for gibbon ape leukemia virus is a novel high affinity sodium-dependent phosphate transporter. J. Biol. Chem..

[67] Miller D.G., Miller A.D. (1994). A family of retroviruses that utilize related phosphate transporters for cell entry. J. Virol..

[68] Matsuda A., Suzuki Y., Honda G., Muramatsu S., Matsuzaki O., Nagano Y., Doi T., Shimotohno K., Harada T., Nishida E. (2003). Large-scale identification and characterization of human genes that activate NF-κB and MAPK signaling pathways. Oncogene.

[69] Demir Karaman E., Işık Z. (2023). Multi-Omics Data Analysis Identifies Prognostic Biomarkers across Cancers. Med. Sci..

[70] Tomczak A., Mortensen J.M., Winnenburg R., Liu C., Alessi D.T., Swamy V., Vallania F., Lofgren S., Haynes W., Shah N.H. (2018). Interpretation of biological experiments changes with evolution of the Gene Ontology and its annotations. Sci. Rep..

[71] Liberti M.V., Locasale J.W. (2016). The Warburg Effect: How Does it Benefit Cancer Cells?. Trends Biochem. Sci..

[72] Pan R., Chen Y. (2021). Management of Oxidative Stress: Crosstalk Between Brown/Beige Adipose Tissues and Skeletal Muscles. Front. Physiol..

[73] Manem V.S.K., Lambie M., Smith I., Smirnov P., Kofia V., Freeman M., Schwartz M.P., Hassanein M., Haibe-Kains B., Bratman S.V. (2019). Modeling cellular response in large-scale radiogenomic databases to advance precision radiotherapy. Cancer Res..

[74] Tsifintaris M., Grigoriadis D., Sitmalidis M., Repanas P., Lazarashvili E.K., Anastasiadi C., Kavakiotis I., Sandaltzopoulos R., Pavlopoulos G.A., Giannakakis A. (2026). ASTRA: A comprehensive resource of stress-induced transcriptional activity in human cell lines. Nucleic Acids Res..

[75] Su D., Xu H., Feng J., Gao Y., Gu L., Ying L., Katsaros D., Yu H., Xu S., Qi M. (2012). PDCD6 is an independent predictor of progression free survival in epithelial ovarian cancer. J. Transl. Med..

[76] Zhang Z., Liu B., Lin Z., Mei L., Chen R., Li Z. (2024). SPP1 could be an immunological and prognostic biomarker: From pan-cancer comprehensive analysis to osteosarcoma validation. FASEB J..

[77] Herbert A.D., Carr A.M., Hoffmann E. (2014). FindFoci: A focus detection algorithm with automated parameter training that closely matches human assignments, reduces human inconsistencies and increases speed of analysis. PLoS ONE.

[78] Ge S.X., Son E.W., Yao R. (2018). iDEP: An integrated web application for differential expression and pathway analysis of RNA-Seq data. BMC Bioinform..

