# Supplementary material for: Differential Radiomodulatory Effects of Sodium Aminodihydrophthalazinedione (Tameron®) on Normal and Cancer Cells Cultures: Antioxidant Activity, DNA Damage Response, and Transcriptomic Profiling
Source: Int J Mol Sci. 2026 Jun 10;27(12):5272. doi: 10.3390/ijms27125272 (PMC13299281; doi:10.3390/ijms27125272)
Supplement: Supplementary file 1 [file ijms-27-05272-s001.zip › ijms-4291986-supplementary.pdf]

Supplementary Materials

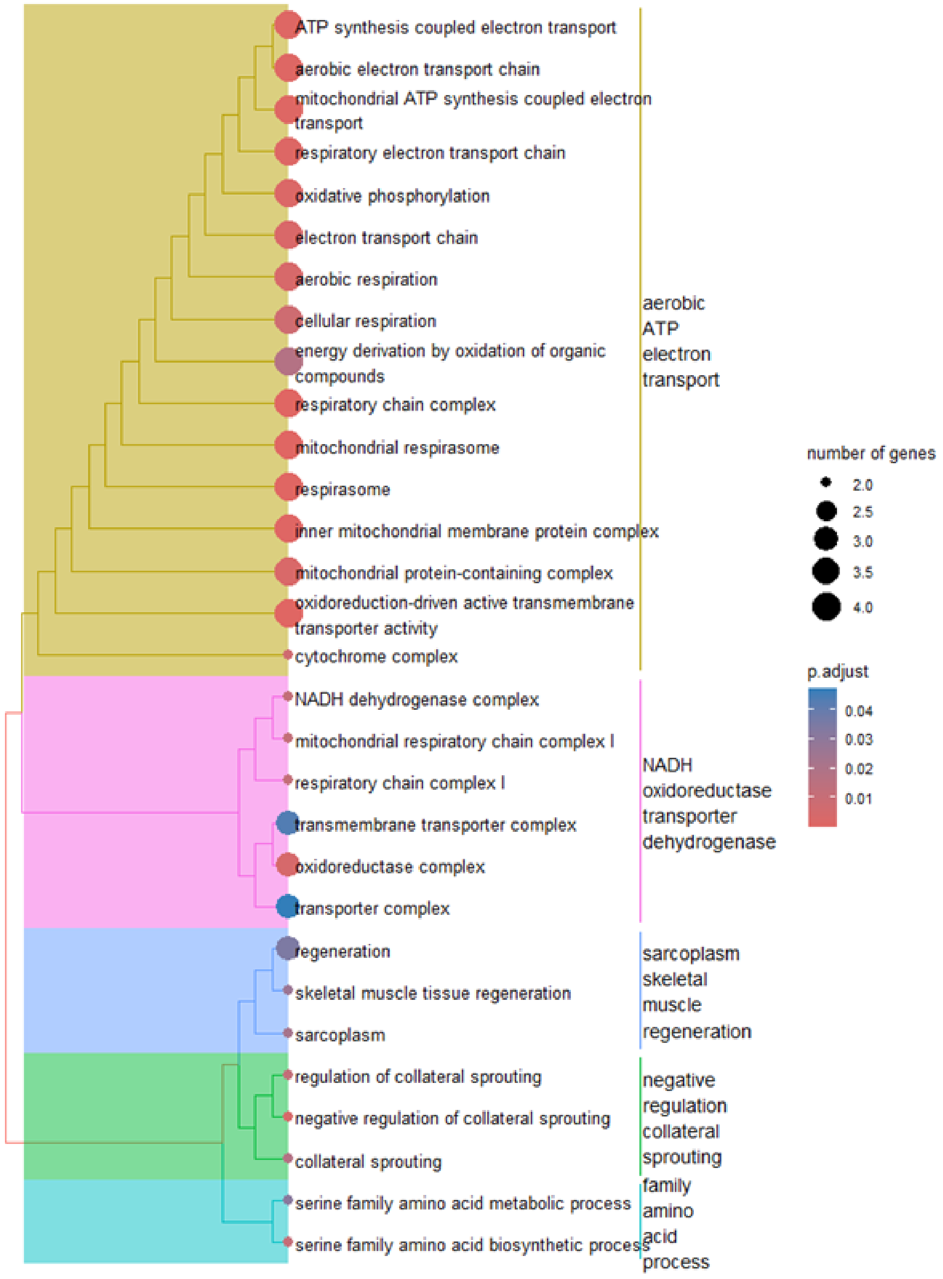

Figure S1. Pathways with decreased enrichment for the 2 mM Tameron® treated group without irradiation (group 1) in comparison to the corresponding control group (cells without irradiation or any drug treatment) after 1 day of incubation.

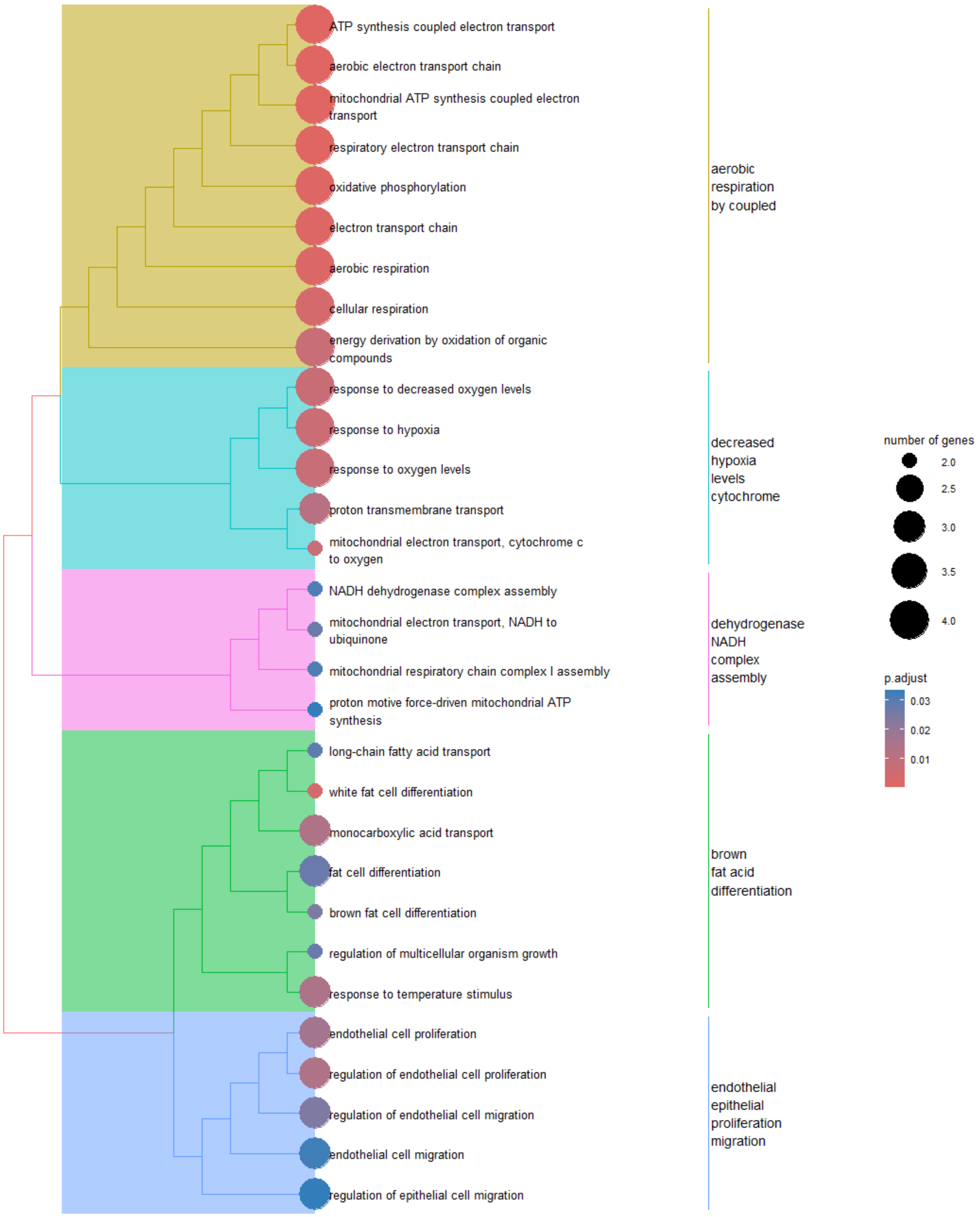

Figure S2. Pathways with increased enrichment for the 2 mM Tameron® treated group without irradiation (group 1) in comparison to the corresponding control group (cells without irradiation or any drug treatment) after 3 days of incubation.

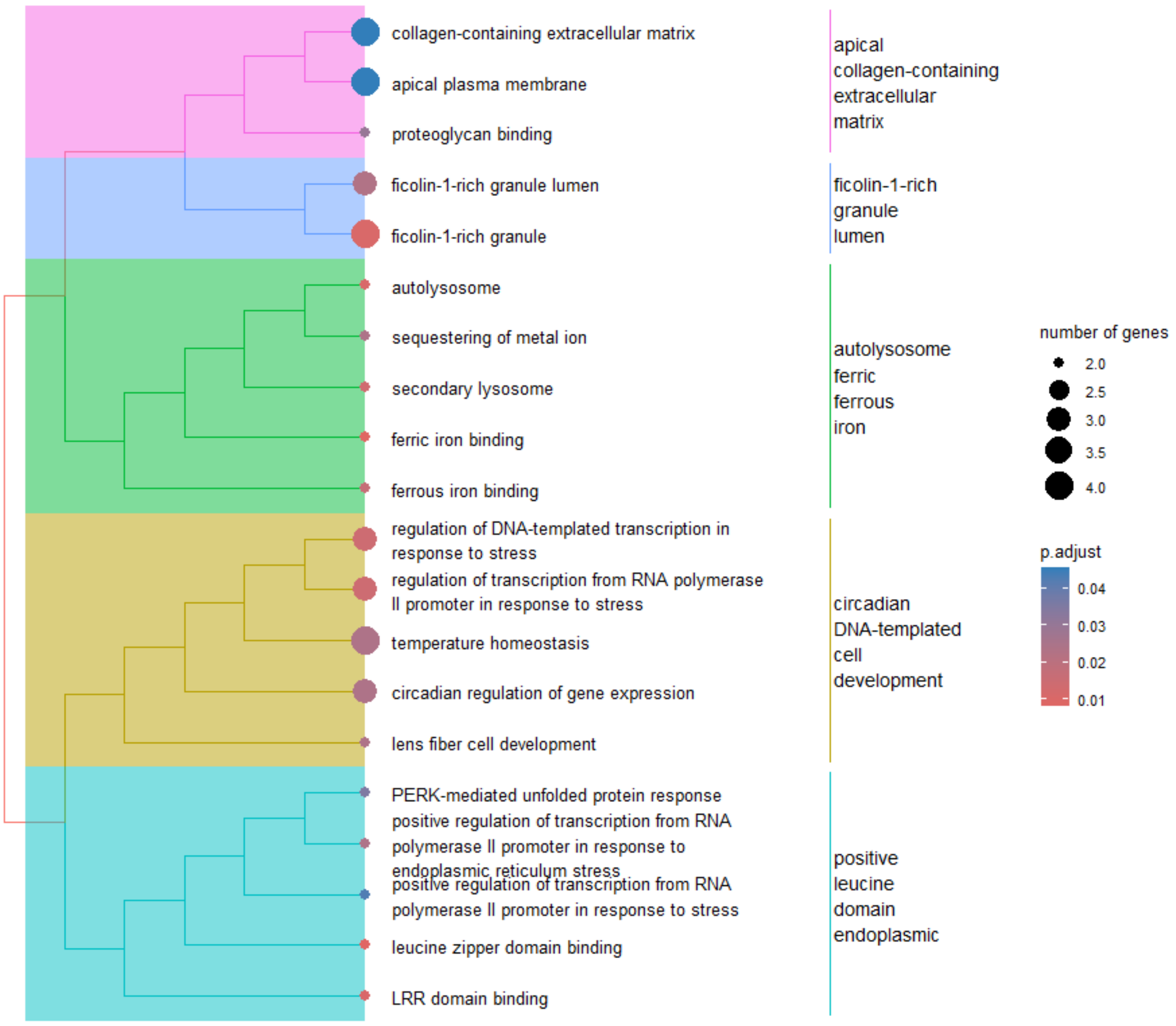

Figure S3. Pathways with decreased enrichment for the 2 mM Tameron® treated group without irradiation (group 1) in comparison to the corresponding control group (cells without irradiation or any drug treatment) after 3 days of incubation.

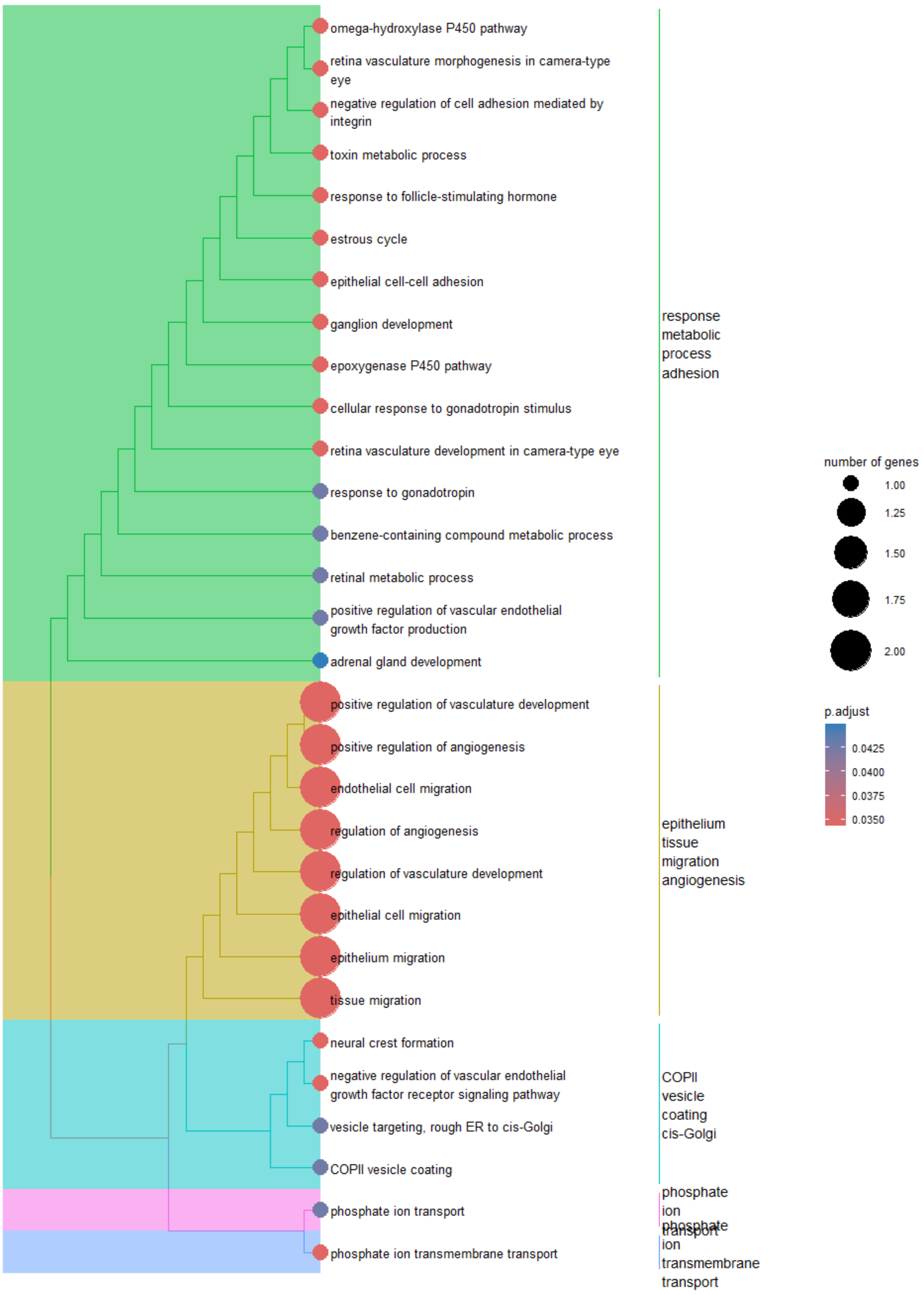

Figure S4. Pathways with increased enrichment for the 2 mM Tameron® treated group with irradiation (group 3) in comparison to the irradiated drug-untreated group (group 2) after 1 day of incubation.

Table S1. Pathways with decreased enrichment for the 2mM Tameron® treated group without irradiation (group 1) vs. control after 1 day of incubation.

ONTOLOGY	ID	Description	GeneRatio	BgRatio	pvalue	p.adjust	qvalue	geneID	Count
BP	GO:0019646	aerobic electron transport chain	4/15	109/21261	8.54e-07	0.000210	0.000134	ENSG00000198888/ENSG00000198763/ENSG00000198712/ENSG00000198727	4
BP	GO:0042773	ATP synthesis coupled electron transport	4/15	117/21261	1.13e-06	0.000210	0.000134	ENSG00000198888/ENSG00000198763/ENSG00000198712/ENSG00000198727	4
BP	GO:0042775	mitochondrial ATP synthesis coupled electron transport	4/15	117/21261	1.13e-06	0.000210	0.000134	ENSG00000198888/ENSG00000198763/ENSG00000198712/ENSG00000198727	4
BP	GO:0022904	respiratory electron transport chain	4/15	141/21261	2.39e-06	0.000332	0.000211	ENSG00000198888/ENSG00000198763/ENSG00000198712/ENSG00000198727	4
BP	GO:0006119	oxidative phosphorylation	4/15	171/21261	5.15e-06	0.000571	0.000364	ENSG00000198888/ENSG00000198763/ENSG00000198712/ENSG00000198727	4
BP	GO:0022900	electron transport chain	4/15	195/21261	8.65e-06	0.000800	0.000510	ENSG00000198888/ENSG00000198763/ENSG00000198712/ENSG00000198727	4
BP	GO:0009060	aerobic respiration	4/15	222/21261	1.44e-05	0.001144	0.000729	ENSG00000198888/ENSG00000198763/ENSG00000198712/ENSG00000198727	4
BP	GO:0045333	cellular respiration	4/15	272/21261	3.20e-05	0.002220	0.001415	ENSG00000198888/ENSG00000198763/ENSG00000198712/ENSG00000198727	4
BP	GO:0015980	energy derivation by oxidation of organic compounds	4/15	369/21261	0.000105	0.006458	0.004115	ENSG00000198888/ENSG00000198763/ENSG00000198712/ENSG00000198727	4
CC	GO:0098803	respiratory chain complex	4/15	107/22568	6.26e-07	2.21e-05	1.38e-05	ENSG00000198888/ENSG00000198763/ENSG00000198712/ENSG00000198727	4
CC	GO:0005746	mitochondrial respirasome	4/15	111/22568	7.26e-07	2.21e-05	1.38e-05	ENSG00000198888/ENSG00000198763/ENSG00000198712/ENSG00000198727	4
CC	GO:0070469	respirasome	4/15	119/22568	9.59e-07	2.21e-05	1.38e-05	ENSG00000198888/ENSG00000198763/ENSG00000198712/ENSG00000198727	4
CC	GO:0098800	inner mitochondrial membrane protein complex	4/15	183/22568	5.33e-06	9.19e-05	5.75e-05	ENSG00000198888/ENSG00000198763/ENSG00000198712/ENSG00000198727	4
CC	GO:0098798	mitochondrial protein-containing complex	4/15	339/22568	5.99e-05	0.000827	0.000517	ENSG00000198888/ENSG00000198763/ENSG00000198712/ENSG00000198727	4
CC	GO:1990204	oxidoreductase complex	3/15	155/22568	0.000136	0.001565	0.000979	ENSG00000198888/ENSG00000198763/ENSG00000198727	3
CC	GO:0070069	cytochrome complex	2/15	42/22568	0.000350	0.003446	0.002155	ENSG00000198712/ENSG00000198727	2
CC	GO:0005747	mitochondrial respiratory chain complex I	2/15	65/22568	0.000837	0.005776	0.003613	ENSG00000198888/ENSG00000198763	2
CC	GO:0030964	NADH dehydrogenase complex	2/15	65/22568	0.000837	0.005776	0.003613	ENSG00000198888/ENSG00000198763	2
CC	GO:0045271	respiratory chain complex I	2/15	65/22568	0.000837	0.005776	0.003613	ENSG00000198888/ENSG00000198763	2
CC	GO:1902495	transmembrane transporter complex	3/15	437/22568	0.002759	0.017305	0.010824	ENSG00000198888/ENSG00000198763/ENSG00000198727	3
CC	GO:1990351	transporter complex	3/15	464/22568	0.003268	0.018792	0.011754	ENSG00000198888/ENSG00000198763/ENSG00000198727	3
CC	GO:0005750	mitochondrial respiratory chain complex III	1/15	13/22568	0.008608	0.042427	0.026537	ENSG00000198727	1
CC	GO:0045275	respiratory chain complex III	1/15	13/22568	0.008608	0.042427	0.026537	ENSG00000198727	1
CC	GO:0044754	autolysosome	1/15	16/22568	0.010585	0.048692	0.030456	ENSG00000087086	1
MF	GO:0015453	oxidoreduction-driven active transmembrane transporter activity	4/15	84/21059	3.11e-07	1.83e-05	8.18e-06	ENSG00000198888/ENSG00000198763/ENSG00000198712/ENSG00000198727	4
MF	GO:0009055	electron transfer activity	4/15	134/21059	2.03e-06	5.98e-05	2.67e-05	ENSG00000198888/ENSG00000198763/ENSG00000198712/ENSG00000198727	4
MF	GO:0015399	primary active transmembrane transporter activity	4/15	204/21059	1.07e-05	0.000211	9.42e-05	ENSG00000198888/ENSG00000198763/ENSG00000198712/ENSG00000198727	4
MF	GO:0008137	NADH dehydrogenase	2/15	56/21059	0.000713	0.006898	0.003077	ENSG00000198888/ENSG00000198763	2

ONTOLOGY	ID	Description	GeneRatio	BgRatio	pvalue	p.adjust	qvalue	geneID	Count
		(ubiquinone) activity							
MF	GO:0050136	NADH dehydrogenase (quinone) activity	2/15	57/21059	0.000739	0.006898	0.003077	ENSG00000198888/ENSG00000198763	2
MF	GO:0003954	NADH dehydrogenase activity	2/15	59/21059	0.000791	0.006898	0.003077	ENSG00000198888/ENSG00000198763	2
MF	GO:0003955	NAD(P)H dehydrogenase (quinone) activity	2/15	60/21059	0.000818	0.006898	0.003077	ENSG00000198888/ENSG00000198763	2
MF	GO:0016655	oxidoreductase activity, acting on NAD(P)H, quinone or similar compound as acceptor	2/15	73/21059	0.001209	0.008914	0.003976	ENSG00000198888/ENSG00000198763	2
MF	GO:0016651	oxidoreductase activity, acting on NAD(P)H	2/15	104/21059	0.002432	0.015944	0.007111	ENSG00000198888/ENSG00000198763	2
MF	GO:0015078	proton transmembrane transporter activity	2/15	156/21059	0.005374	0.031706	0.014142	ENSG00000198712/ENSG00000198727	2
MF	GO:0008199	ferric iron binding	1/15	10/21059	0.007102	0.037609	0.016775	ENSG00000087086	1
MF	GO:0004859	phospholipase inhibitor activity	1/15	12/21059	0.008516	0.037609	0.016775	ENSG00000135046	1
MF	GO:0042301	phosphate ion binding	1/15	13/21059	0.009223	0.037609	0.016775	ENSG00000065911	1
MF	GO:0055102	lipase inhibitor activity	1/15	16/21059	0.011340	0.037609	0.016775	ENSG00000135046	1
MF	GO:0016846	carbon-sulfur lyase activity	1/15	17/21059	0.012045	0.037609	0.016775	ENSG00000116761	1
MF	GO:0004129	cytochrome-c oxidase activity	1/15	18/21059	0.012749	0.037609	0.016775	ENSG00000198712	1
MF	GO:0016646	oxidoreductase activity, acting on the CH-NH group of donors, NAD or NADP as acceptor	1/15	18/21059	0.012749	0.037609	0.016775	ENSG00000065911	1
MF	GO:0016675	oxidoreductase activity, acting on a heme group of donors	1/15	18/21059	0.012749	0.037609	0.016775	ENSG00000198712	1
MF	GO:0019966	interleukin-1 binding	1/15	18/21059	0.012749	0.037609	0.016775	ENSG00000143575	1
MF	GO:0098641	cadherin binding involved in cell-cell adhesion	1/15	18/21059	0.012749	0.037609	0.016775	ENSG00000135046	1
MF	GO:0022853	active monoatomic ion transmembrane transporter activity	2/15	262/21059	0.014551	0.040881	0.018234	ENSG00000198712/ENSG00000198727	2
MF	GO:0031681	G-protein beta-subunit binding	1/15	23/21059	0.016263	0.043615	0.019454	ENSG00000127920	1
MF	GO:0008198	ferrous iron binding	1/15	26/21059	0.018366	0.047113	0.021014	ENSG00000087086	1

Table S2. Pathways with increased enrichment for the 2mM Tameron® treated group without irradiation (group 1) vs. control after 3 days of incubation.

ONTOLOGY	ID	Description	GeneRatio	BgRatio	pvalue	p.adjust	qvalue	geneID	Count
BP	GO:0019646	aerobic electron transport chain	4/17	109/21288	1.47e-06	0.000330	0.000207	ENSG00000198804/ENSG00000198712/ENSG00000198886/ENSG00000198695	4
BP	GO:0042773	ATP synthesis coupled electron transport	4/17	117/21288	1.95e-06	0.000330	0.000207	ENSG00000198804/ENSG00000198712/ENSG00000198886/ENSG00000198695	4
BP	GO:0042775	mitochondrial ATP synthesis coupled electron transport	4/17	117/21288	1.95e-06	0.000330	0.000207	ENSG00000198804/ENSG00000198712/ENSG00000198886/ENSG00000198695	4
BP	GO:0022904	respiratory electron transport chain	4/17	140/21288	3.99e-06	0.000507	0.000317	ENSG00000198804/ENSG00000198712/ENSG00000198886/ENSG00000198695	4
BP	GO:0006119	oxidative phosphorylation	4/17	171/21288	8.82e-06	0.000896	0.000561	ENSG00000198804/ENSG00000198712/ENSG00000198886/ENSG00000198695	4
BP	GO:0022900	electron transport chain	4/17	194/21288	1.45e-05	0.001228	0.000768	ENSG00000198804/ENSG00000198712/ENSG00000198886/ENSG00000198695	4
BP	GO:0009060	aerobic respiration	4/17	224/21288	2.55e-05	0.001851	0.001158	ENSG00000198804/ENSG00000198712/ENSG00000198886/ENSG00000198695	4
BP	GO:0045333	cellular respiration	4/17	274/21288	5.60e-05	0.003556	0.002225	ENSG00000198804/ENSG00000198712/ENSG00000198886/ENSG00000198695	4
BP	GO:0050872	white fat cell differentiation	2/17	17/21288	8.11e-05	0.004575	0.002863	ENSG00000132170/ENSG00000170323	2
BP	GO:0001666	response to hypoxia	4/17	344/21288	0.000135	0.006859	0.004292	ENSG00000132170/ENSG00000198804/ENSG00000198712/ENSG00000198886	4
BP	GO:0036293	response to decreased oxygen levels	4/17	360/21288	0.000161	0.007426	0.004647	ENSG00000132170/ENSG00000198804/ENSG00000198712/ENSG00000198886	4
BP	GO:0015980	energy derivation by oxidation of organic compounds	4/17	370/21288	0.000179	0.007537	0.004717	ENSG00000198804/ENSG00000198712/ENSG00000198886/ENSG00000198695	4
BP	GO:0006123	mitochondrial electron transport, cytochrome c to oxygen	2/17	26/21288	0.000193	0.007537	0.004717	ENSG00000198804/ENSG00000198712	2
BP	GO:0070482	response to oxygen levels	4/17	387/21288	0.000212	0.007698	0.004818	ENSG00000132170/ENSG00000198804/ENSG00000198712/ENSG00000198886	4
BP	GO:1902600	proton transmembrane transport	3/17	177/21288	0.000353	0.011944	0.007474	ENSG00000198804/ENSG00000198712/ENSG00000198886	3
BP	GO:0001936	regulation of endothelial cell proliferation	3/17	193/21288	0.000454	0.013990	0.008755	ENSG00000132170/ENSG00000249915/ENSG00000113140	3
BP	GO:0015718	monocarboxylic acid transport	3/17	195/21288	0.000468	0.013990	0.008755	ENSG00000132170/ENSG00000170323/ENSG00000108932	3
BP	GO:0009266	response to temperature stimulus	3/17	202/21288	0.000519	0.014644	0.009164	ENSG00000145287/ENSG00000249915/ENSG00000204520	3
BP	GO:0001935	endothelial cell proliferation	3/17	215/21288	0.000622	0.016636	0.010411	ENSG00000132170/ENSG00000249915/ENSG00000113140	3
BP	GO:0050873	brown fat cell differentiation	2/17	58/21288	0.000966	0.024398	0.015268	ENSG00000145287/ENSG00000170323	2
BP	GO:0010594	regulation of endothelial cell migration	3/17	254/21288	0.001009	0.024398	0.015268	ENSG00000132170/ENSG00000249915/ENSG00000113140	3
BP	GO:0006120	mitochondrial electron transport, NADH to ubiquinone	2/17	66/21288	0.001249	0.027688	0.017326	ENSG00000198886/ENSG00000198695	2
BP	GO:0040014	regulation of multicellular organism growth	2/17	67/21288	0.001287	0.027688	0.017326	ENSG00000145287/ENSG00000166794	2
BP	GO:0045444	fat cell differentiation	3/17	278/21288	0.001308	0.027688	0.017326	ENSG00000132170/ENSG00000145287/ENSG00000170323	3
BP	GO:0015909	long-chain fatty acid transport	2/17	71/21288	0.001444	0.029343	0.018362	ENSG00000132170/ENSG00000170323	2
BP	GO:0010257	NADH dehydrogenase complex assembly	2/17	76/21288	0.001652	0.031087	0.019453	ENSG00000198886/ENSG00000198695	2

ONTOLOGY	ID	Description	GeneRatio	BgRatio	pvalue	p.adjust	qvalue	geneID	Count
BP	GO:0032981	mitochondrial respiratory chain complex I assembly	2/17	76/21288	0.001652	0.031087	0.019453	ENSG00000198886/ENSG00000198695	2
BP	GO:0043542	endothelial cell migration	3/17	310/21288	0.001787	0.032428	0.020292	ENSG00000132170/ENSG00000249915/ENSG00000113140	3
BP	GO:0042776	proton motive force-driven mitochondrial ATP synthesis	2/17	82/21288	0.001920	0.033432	0.020921	ENSG00000198886/ENSG00000198695	2
BP	GO:0010632	regulation of epithelial cell migration	3/17	321/21288	0.001974	0.033432	0.020921	ENSG00000132170/ENSG00000249915/ENSG00000113140	3
BP	GO:0001937	negative regulation of endothelial cell proliferation	2/17	86/21288	0.002109	0.034561	0.021627	ENSG00000132170/ENSG00000113140	2
BP	GO:0015986	proton motive force-driven ATP synthesis	2/17	91/21288	0.002357	0.037423	0.023419	ENSG00000198886/ENSG00000198695	2
BP	GO:0045765	regulation of angiogenesis	3/17	386/21288	0.003330	0.045774	0.028645	ENSG00000132170/ENSG00000249915/ENSG00000113140	3
BP	GO:0010038	response to metal ion	3/17	387/21288	0.003354	0.045774	0.028645	ENSG00000249915/ENSG00000170323/ENSG00000198804	3
BP	GO:0021549	cerebellum development	2/17	109/21288	0.003360	0.045774	0.028645	ENSG00000198804/ENSG00000198886	2
BP	GO:0046942	carboxylic acid transport	3/17	389/21288	0.003403	0.045774	0.028645	ENSG00000132170/ENSG00000170323/ENSG00000108932	3
BP	GO:0015849	organic acid transport	3/17	390/21288	0.003428	0.045774	0.028645	ENSG00000132170/ENSG00000170323/ENSG00000108932	3
BP	GO:1901342	regulation of vasculature development	3/17	395/21288	0.003553	0.045774	0.028645	ENSG00000132170/ENSG00000249915/ENSG00000113140	3
BP	GO:0120162	positive regulation of cold-induced thermogenesis	2/17	113/21288	0.003605	0.045774	0.028645	ENSG00000145287/ENSG00000170323	2
BP	GO:0010631	epithelial cell migration	3/17	403/21288	0.003759	0.045774	0.028645	ENSG00000132170/ENSG00000249915/ENSG00000113140	3
BP	GO:0090132	epithelium migration	3/17	406/21288	0.003838	0.045774	0.028645	ENSG00000132170/ENSG00000249915/ENSG00000113140	3
BP	GO:0006754	ATP biosynthetic process	2/17	117/21288	0.003859	0.045774	0.028645	ENSG00000198886/ENSG00000198695	2
BP	GO:0033108	mitochondrial respiratory chain complex assembly	2/17	118/21288	0.003924	0.045774	0.028645	ENSG00000198886/ENSG00000198695	2
BP	GO:0090130	tissue migration	3/17	411/21288	0.003972	0.045774	0.028645	ENSG00000132170/ENSG00000249915/ENSG00000113140	3
BP	GO:0022037	metencephalon development	2/17	120/21288	0.004055	0.045774	0.028645	ENSG00000198804/ENSG00000198886	2
BP	GO:0015908	fatty acid transport	2/17	122/21288	0.004188	0.046247	0.028941	ENSG00000132170/ENSG00000170323	2
BP	GO:0009206	purine ribonucleoside triphosphate biosynthetic process	2/17	127/21288	0.004529	0.047676	0.029834	ENSG00000198886/ENSG00000198695	2
BP	GO:0050678	regulation of epithelial cell proliferation	3/17	432/21288	0.004567	0.047676	0.029834	ENSG00000132170/ENSG00000249915/ENSG00000113140	3
BP	GO:0009145	purine nucleoside triphosphate biosynthetic process	2/17	128/21288	0.004599	0.047676	0.029834	ENSG00000198886/ENSG00000198695	2
BP	GO:0043280	positive regulation of cysteine-type endopeptidase activity involved in apoptotic process	2/17	132/21288	0.004883	0.048404	0.030290	ENSG00000132170/ENSG00000249915	2
BP	GO:0009201	ribonucleoside triphosphate biosynthetic process	2/17	133/21288	0.004955	0.048404	0.030290	ENSG00000198886/ENSG00000198695	2
BP	GO:0009408	response to heat	2/17	133/21288	0.004955	0.048404	0.030290	ENSG00000249915/ENSG00000204520	2

ONTOLOGY	ID	Description	GeneRatio	BgRatio	pvalue	p.adjust	qvalue	geneID	Count
CC	GO:0098803	respiratory chain complex	4/17	110/22585	1.21e-06	4.14e-05	2.44e-05	ENSG00000198804/ENSG00000198712/ENSG00000198886/ENSG00000198695	4
CC	GO:0005746	mitochondrial respirasome	4/17	114/22585	1.39e-06	4.14e-05	2.44e-05	ENSG00000198804/ENSG00000198712/ENSG00000198886/ENSG00000198695	4
CC	GO:0070469	respirasome	4/17	122/22585	1.83e-06	4.14e-05	2.44e-05	ENSG00000198804/ENSG00000198712/ENSG00000198886/ENSG00000198695	4
CC	GO:1990204	oxidoreductase complex	4/17	158/22585	5.11e-06	8.69e-05	5.11e-05	ENSG00000103018/ENSG00000198804/ENSG00000198886/ENSG00000198695	4
CC	GO:0098800	inner mitochondrial membrane protein complex	4/17	187/22585	9.96e-06	0.000135	7.96e-05	ENSG00000198804/ENSG00000198712/ENSG00000198886/ENSG00000198695	4
CC	GO:0098798	mitochondrial protein-containing complex	4/17	342/22585	0.000105	0.001192	0.000701	ENSG00000198804/ENSG00000198712/ENSG00000198886/ENSG00000198695	4
CC	GO:0005751	mitochondrial respiratory chain complex IV	2/17	26/22585	0.000171	0.001666	0.000980	ENSG00000198804/ENSG00000198712	2
CC	GO:0045277	respiratory chain complex IV	2/17	29/22585	0.000214	0.001818	0.001070	ENSG00000198804/ENSG00000198712	2
CC	GO:0070069	cytochrome complex	2/17	43/22585	0.000473	0.003573	0.002102	ENSG00000198804/ENSG00000198712	2
CC	GO:0005747	mitochondrial respiratory chain complex I	2/17	67/22585	0.001146	0.006492	0.003819	ENSG00000198886/ENSG00000198695	2
CC	GO:0030964	NADH dehydrogenase complex	2/17	67/22585	0.001146	0.006492	0.003819	ENSG00000198886/ENSG00000198695	2
CC	GO:0045271	respiratory chain complex I	2/17	67/22585	0.001146	0.006492	0.003819	ENSG00000198886/ENSG00000198695	2
CC	GO:1902495	transmembrane transporter complex	3/17	489/22585	0.005472	0.028621	0.016836	ENSG00000198804/ENSG00000198886/ENSG00000198695	3
CC	GO:0140534	endoplasmic reticulum protein-containing complex	2/17	156/22585	0.006023	0.029254	0.017208	ENSG00000166794/ENSG00000166562	2
CC	GO:0034663	endoplasmic reticulum chaperone complex	1/17	12/22585	0.008997	0.040788	0.023993	ENSG00000166794	1
CC	GO:0005750	mitochondrial respiratory chain complex III	1/17	14/22585	0.010490	0.041958	0.024681	ENSG00000198804	1
CC	GO:0045275	respiratory chain complex III	1/17	14/22585	0.010490	0.041958	0.024681	ENSG00000198804	1
CC	GO:0030127	COPII vesicle coat	1/17	17/22585	0.012724	0.048068	0.028275	ENSG00000249915	1
MF	GO:0015453	oxidoreduction-driven active transmembrane transporter activity	4/17	84/21092	5.35e-07	4.07e-05	1.47e-05	ENSG00000198804/ENSG00000198712/ENSG00000198886/ENSG00000198695	4
MF	GO:0009055	electron transfer activity	4/17	134/21092	3.48e-06	0.000132	4.76e-05	ENSG00000198804/ENSG00000198712/ENSG00000198886/ENSG00000198695	4
MF	GO:0015399	primary active transmembrane transporter activity	4/17	205/21092	1.87e-05	0.000473	0.000170	ENSG00000198804/ENSG00000198712/ENSG00000198886/ENSG00000198695	4
MF	GO:0036041	long-chain fatty acid binding	2/17	17/21092	8.26e-05	0.001314	0.000473	ENSG00000132170/ENSG00000170323	2
MF	GO:0004129	cytochrome-c oxidase activity	2/17	18/21092	9.28e-05	0.001314	0.000473	ENSG00000198804/ENSG00000198712	2
MF	GO:0016675	oxidoreductase activity, acting on a heme group of donors	2/17	19/21092	0.000104	0.001314	0.000473	ENSG00000198804/ENSG00000198712	2
MF	GO:0005504	fatty acid binding	2/17	51/21092	0.000762	0.007275	0.002620	ENSG00000132170/ENSG00000170323	2
MF	GO:0008137	NADH dehydrogenase (ubiquinone) activity	2/17	56/21092	0.000918	0.007275	0.002620	ENSG00000198886/ENSG00000198695	2
MF	GO:0050136	NADH dehydrogenase (quinone) activity	2/17	57/21092	0.000951	0.007275	0.002620	ENSG00000198886/ENSG00000198695	2

ONTOLOGY	ID	Description	GeneRatio	BgRatio	pvalue	p.adjust	qvalue	geneID	Count
MF	GO:0003954	NADH dehydrogenase activity	2/17	59/21092	0.001018	0.007275	0.002620	ENSG00000198886/ENSG00000198695	2
MF	GO:0003955	NAD(P)H dehydrogenase (quinone) activity	2/17	60/21092	0.001053	0.007275	0.002620	ENSG00000198886/ENSG00000198695	2
MF	GO:0016655	oxidoreductase activity, acting on NAD(P)H, quinone or similar compound as acceptor	2/17	73/21092	0.001554	0.009840	0.003544	ENSG00000198886/ENSG00000198695	2
MF	GO:0033293	monocarboxylic acid binding	2/17	84/21092	0.002050	0.011986	0.004316	ENSG00000132170/ENSG00000170323	2
MF	GO:0005518	collagen binding	2/17	93/21092	0.002505	0.013601	0.004898	ENSG00000113140/ENSG00000166794	2
MF	GO:0016651	oxidoreductase activity, acting on NAD(P)H	2/17	103/21092	0.003062	0.015513	0.005587	ENSG00000198886/ENSG00000198695	2
MF	GO:0020037	heme binding	2/17	156/21092	0.006873	0.031490	0.011340	ENSG00000103018/ENSG00000198804	2
MF	GO:0015078	proton transmembrane transporter activity	2/17	158/21092	0.007044	0.031490	0.011340	ENSG00000198804/ENSG00000198712	2
MF	GO:0046906	tetrapyrrole binding	2/17	166/21092	0.007748	0.032130	0.011570	ENSG00000103018/ENSG00000198804	2
MF	GO:0004955	prostaglandin receptor activity	1/17	10/21092	0.008032	0.032130	0.011570	ENSG00000132170	1
MF	GO:0004954	prostanoid receptor activity	1/17	11/21092	0.008832	0.033563	0.012086	ENSG00000132170	1
MF	GO:0097677	STAT family protein binding	1/17	12/21092	0.009632	0.033708	0.012138	ENSG00000132170	1
MF	GO:0004252	serine-type endopeptidase activity	2/17	202/21092	0.011292	0.033708	0.012138	ENSG00000196611/ENSG00000166562	2
MF	GO:0031406	carboxylic acid binding	2/17	203/21092	0.011399	0.033708	0.012138	ENSG00000132170/ENSG00000170323	2
MF	GO:0016661	oxidoreductase activity, acting on other nitrogenous compounds as donors	1/17	15/21092	0.012026	0.033708	0.012138	ENSG00000103018	1
MF	GO:0043177	organic acid binding	2/17	215/21092	0.012718	0.033708	0.012138	ENSG00000132170/ENSG00000170323	2
MF	GO:0005324	long-chain fatty acid transporter activity	1/17	16/21092	0.012823	0.033708	0.012138	ENSG00000170323	1
MF	GO:0048038	quinone binding	1/17	16/21092	0.012823	0.033708	0.012138	ENSG00000198886	1
MF	GO:0008236	serine-type peptidase activity	2/17	220/21092	0.013286	0.033708	0.012138	ENSG00000196611/ENSG00000166562	2
MF	GO:0004953	icosanoid receptor activity	1/17	17/21092	0.013619	0.033708	0.012138	ENSG00000132170	1
MF	GO:0046965	nuclear retinoid X receptor binding	1/17	17/21092	0.013619	0.033708	0.012138	ENSG00000132170	1
MF	GO:0017171	serine hydrolase activity	2/17	224/21092	0.013749	0.033708	0.012138	ENSG00000196611/ENSG00000166562	2
MF	GO:0016018	cyclosporin A binding	1/17	22/21092	0.017591	0.041326	0.014882	ENSG00000166794	1
MF	GO:0031681	G-protein beta-subunit binding	1/17	23/21092	0.018384	0.041326	0.014882	ENSG00000127920	1
MF	GO:0022853	active monoatomic ion transmembrane transporter activity	2/17	262/21092	0.018488	0.041326	0.014882	ENSG00000198804/ENSG00000198712	2
MF	GO:0046703	natural killer cell lectin-like receptor binding	1/17	25/21092	0.019967	0.043358	0.015614	ENSG00000204520	1
MF	GO:0070412	R-SMAD binding	1/17	26/21092	0.020758	0.043823	0.015781	ENSG00000132170	1
MF	GO:0042974	nuclear retinoic acid receptor binding	1/17	28/21092	0.022338	0.045884	0.016523	ENSG00000132170	1

Table S3. Pathways with decreased enrichment for the 2mM Tameron® treated group without irradiation (group 1) vs. control after 3 days of incubation.

ONTOLOGY	ID	Description	GeneRatio	BgRatio	pvalue	p.adjust	qvalue	geneID	Count
BP	GO:0043618	regulation of transcription from RNA polymerase II promoter in response to stress	3/28	37/21261	1.54e-05	0.014515	0.011043	ENSG00000120738/ENSG00000175197/ENSG00000128272	3
BP	GO:0043620	regulation of DNA-templated transcription in response to stress	3/28	44/21261	2.61e-05	0.014515	0.011043	ENSG00000120738/ENSG00000175197/ENSG00000128272	3
BP	GO:0051238	sequestering of metal ion	2/28	11/21261	9.13e-05	0.023921	0.018200	ENSG00000167996/ENSG00000087086	2
BP	GO:0032922	circadian regulation of gene expression	3/28	71/21261	0.000110	0.023921	0.018200	ENSG00000120738/ENSG00000105835/ENSG00000128272	3
BP	GO:0001659	temperature homeostasis	4/28	206/21261	0.000146	0.023921	0.018200	ENSG00000120738/ENSG00000175197/ENSG00000087460/ENSG00000128272	4
BP	GO:0070307	lens fiber cell development	2/28	14/21261	0.000151	0.023921	0.018200	ENSG00000026025/ENSG00000128272	2
BP	GO:1990440	positive regulation of transcription from RNA pol II promoter in response to ER stress	2/28	14/21261	0.000151	0.023921	0.018200	ENSG00000175197/ENSG00000128272	2
BP	GO:0036499	PERK-mediated unfolded protein response	2/28	18/21261	0.000253	0.035077	0.026687	ENSG00000175197/ENSG00000128272	2
BP	GO:0036003	positive regulation of transcription from RNA pol II promoter in response to stress	2/28	21/21261	0.000346	0.042691	0.032480	ENSG00000175197/ENSG00000128272	2
CC	GO:0044754	autolysosome	2/28	16/22568	0.000176	0.010604	0.008219	ENSG00000167996/ENSG00000087086	2
CC	GO:0101002	ficolin-1-rich granule	4/28	235/22568	0.000193	0.010604	0.008219	ENSG00000143106/ENSG00000164733/ENSG00000167996/ENSG00000131981	4
CC	GO:0005767	secondary lysosome	2/28	23/22568	0.000370	0.013550	0.010503	ENSG00000167996/ENSG00000087086	2
CC	GO:1904813	ficolin-1-rich granule lumen	3/28	150/22568	0.000835	0.022951	0.017790	ENSG00000143106/ENSG00000164733/ENSG00000167996	3
CC	GO:0016324	apical plasma membrane	4/28	444/22568	0.002081	0.045473	0.035247	ENSG00000115414/ENSG00000164733/ENSG00000135046/ENSG00000087460	4
CC	GO:0062023	collagen-containing extracellular matrix	4/28	466/22568	0.002480	0.045473	0.035247	ENSG00000115414/ENSG00000164733/ENSG00000135046/ENSG00000131981	4
MF	GO:0008199	ferric iron binding	2/28	10/21059	7.62e-05	0.008038	0.005756	ENSG00000167996/ENSG00000087086	2
MF	GO:0043522	leucine zipper domain binding	2/28	13/21059	0.000132	0.008038	0.005756	ENSG00000175197/ENSG00000128272	2
MF	GO:0030275	LRR domain binding	2/28	18/21059	0.000257	0.010468	0.007497	ENSG00000175197/ENSG00000128272	2
MF	GO:0008198	ferrous iron binding	2/28	26/21059	0.000543	0.016568	0.011865	ENSG00000167996/ENSG00000087086	2
MF	GO:0043394	proteoglycan binding	2/28	39/21059	0.001225	0.029899	0.021412	ENSG00000115414/ENSG00000164733	2

Table S4. Pathways with increased enrichment for the 2mM Tameron® treated group with irradiation (group 3) vs. irradiated drug-untreated group (group 2) after 1 day of incubation.

ONTOLOGY	ID	Description	GeneRatio	BgRatio	RichFactor	FoldEnrich	zScore	pvalue	p.adjust	qvalue	geneID	Count
BP	GO:0045766	positive regulation of angiogenesis	2/4	197/21288	0.010152	54.030	10.251	0.000505	0.034264	0.008974	ENSG00000138061/ENSG00000249915	2
BP	GO:1904018	positive regulation of vasculature development	2/4	200/21288	0.01000	53.220	10.172	0.000520	0.034264	0.008974	ENSG00000138061/ENSG00000249915	2
BP	GO:0043542	endothelial cell migration	2/4	310/21288	0.006452	34.335	8.105	0.001244	0.034264	0.008974	ENSG00000138061/ENSG00000249915	2
BP	GO:0061299	retina vasculature morphogenesis in camera-type eye	1/4	10/21288	0.10000	532.200	23.033	0.001878	0.034264	0.008974	ENSG00000138061	1
BP	GO:0097267	omega-hydroxylase P450 pathway	1/4	10/21288	0.10000	532.200	23.033	0.001878	0.034264	0.008974	ENSG00000138061	1
BP	GO:0045765	regulation of angiogenesis	2/4	386/21288	0.005181	27.575	7.223	0.001921	0.034264	0.008974	ENSG00000138061/ENSG00000249915	2
BP	GO:1901342	regulation of vasculature development	2/4	395/21288	0.005063	26.947	7.136	0.002010	0.034264	0.008974	ENSG00000138061/ENSG00000249915	2
BP	GO:0010631	epithelial cell migration	2/4	403/21288	0.004963	26.412	7.060	0.002092	0.034264	0.008974	ENSG00000138061/ENSG00000249915	2
BP	GO:0090132	epithelium migration	2/4	406/21288	0.004926	26.217	7.033	0.002122	0.034264	0.008974	ENSG00000138061/ENSG00000249915	2
BP	GO:0090130	tissue migration	2/4	411/21288	0.004866	25.898	6.987	0.002174	0.034264	0.008974	ENSG00000138061/ENSG00000249915	2
BP	GO:0030948	negative regulation of VEGF receptor signaling pathway	1/4	12/21288	0.08333	443.500	21.019	0.002253	0.034264	0.008974	ENSG00000249915	1
BP	GO:0033629	negative regulation of cell adhesion mediated by integrin	1/4	12/21288	0.08333	443.500	21.019	0.002253	0.034264	0.008974	ENSG00000138061	1
BP	GO:0009404	toxin metabolic process	1/4	15/21288	0.06667	354.800	18.791	0.002816	0.034264	0.008974	ENSG00000138061	1
BP	GO:0014029	neural crest formation	1/4	16/21288	0.06250	332.625	18.191	0.003003	0.034264	0.008974	ENSG00000249915	1
BP	GO:0032354	response to follicle-stimulating hormone	1/4	16/21288	0.06250	332.625	18.191	0.003003	0.034264	0.008974	ENSG00000138061	1
BP	GO:0044849	estrous cycle	1/4	16/21288	0.06250	332.625	18.191	0.003003	0.034264	0.008974	ENSG00000138061	1
BP	GO:0090136	epithelial cell-cell adhesion	1/4	16/21288	0.06250	332.625	18.191	0.003003	0.034264	0.008974	ENSG00000138061	1
BP	GO:0035435	phosphate ion transmembrane transport	1/4	17/21288	0.05882	313.059	17.645	0.003191	0.034264	0.008974	ENSG00000144136	1
BP	GO:0061548	ganglion development	1/4	17/21288	0.05882	313.059	17.645	0.003191	0.034264	0.008974	ENSG00000138061	1
BP	GO:0019373	epoxygenase P450 pathway	1/4	18/21288	0.05556	295.667	17.145	0.003378	0.034264	0.008974	ENSG00000138061	1
BP	GO:0071371	cellular response to gonadotropin stimulus	1/4	18/21288	0.05556	295.667	17.145	0.003378	0.034264	0.008974	ENSG00000138061	1
BP	GO:0061298	retina vasculature development in camera-type eye	1/4	19/21288	0.05263	280.105	16.685	0.003566	0.034521	0.009042	ENSG00000138061	1
BP	GO:0034698	response to gonadotropin	1/4	28/21288	0.03571	190.071	13.724	0.005251	0.042794	0.011209	ENSG00000138061	1
BP	GO:0042537	benzene-containing compound metabolic process	1/4	28/21288	0.03571	190.071	13.724	0.005251	0.042794	0.011209	ENSG00000138061	1
BP	GO:0006817	phosphate ion transport	1/4	29/21288	0.03448	183.517	13.483	0.005438	0.042794	0.011209	ENSG00000144136	1
BP	GO:0048207	vesicle targeting rough ER to cis-Golgi	1/4	29/21288	0.03448	183.517	13.483	0.005438	0.042794	0.011209	ENSG00000249915	1
BP	GO:0048208	COPII vesicle coating	1/4	29/21288	0.03448	183.517	13.483	0.005438	0.042794	0.011209	ENSG00000249915	1
BP	GO:0042574	retinal metabolic process	1/4	30/21288	0.03333	177.400	13.254	0.005625	0.042794	0.011209	ENSG00000138061	1
BP	GO:0010575	positive regulation of vascular endothelial growth factor production	1/4	32/21288	0.03125	166.313	12.829	0.006000	0.044066	0.011542	ENSG00000138061	1

ONTOLOGY	ID	Description	GeneRatio	BgRatio	RichFactor	FoldEnrich	zScore	pvalue	p.adjust	qvalue	geneID	Count
BP	GO:0030325	adrenal gland development	1/4	34/21288	0.02941	156.529	12.442	0.006374	0.044914	0.011764	ENSG00000138061	1
BP	GO:0006901	vesicle coating	1/4	42/21288	0.02381	126.714	11.180	0.007869	0.049025	0.012841	ENSG00000249915	1
CC	GO:0030127	COPII vesicle coat	1/4	17/22585	0.05882	332.132	18.178	0.003008	0.029422	0.006194	ENSG00000249915	1
CC	GO:0005834	heterotrimeric G-protein complex	1/4	35/22585	0.02857	161.321	12.633	0.006185	0.029422	0.006194	ENSG00000127920	1
CC	GO:0031463	Cul3-RING ubiquitin ligase complex	1/4	39/22585	0.02564	144.776	11.960	0.006890	0.029422	0.006194	ENSG00000249915	1
CC	GO:1905360	GTPase complex	1/4	42/22585	0.02381	134.435	11.520	0.007418	0.029422	0.006194	ENSG00000127920	1
CC	GO:0070971	endoplasmic reticulum exit site	1/4	44/22585	0.02273	128.324	11.251	0.007771	0.029422	0.006194	ENSG00000249915	1
CC	GO:0031234	extrinsic component of cytoplasmic side of plasma membrane	1/4	50/22585	0.02000	112.925	10.545	0.008827	0.029422	0.006194	ENSG00000127920	1
CC	GO:0030120	vesicle coat	1/4	68/22585	0.01471	83.033	9.017	0.011990	0.034257	0.007212	ENSG00000249915	1
CC	GO:0030117	membrane coat	1/4	103/22585	0.00971	54.818	7.286	0.018119	0.037286	0.007850	ENSG00000249915	1
CC	GO:0048475	coated membrane	1/4	103/22585	0.00971	54.818	7.286	0.018119	0.037286	0.007850	ENSG00000249915	1
CC	GO:0019897	extrinsic component of plasma membrane	1/4	106/22585	0.00943	53.267	7.179	0.018643	0.037286	0.007850	ENSG00000127920	1
CC	GO:0012507	ER to Golgi transport vesicle membrane	1/4	156/22585	0.00641	36.194	5.871	0.027346	0.045458	0.009570	ENSG00000249915	1
CC	GO:0009898	cytoplasmic side of plasma membrane	1/4	175/22585	0.00571	32.264	5.526	0.030638	0.045458	0.009570	ENSG00000127920	1
CC	GO:0019898	extrinsic component of membrane	1/4	185/22585	0.00541	30.520	5.366	0.032367	0.045458	0.009570	ENSG00000127920	1
CC	GO:0030134	COPII-coated ER to Golgi transport vesicle	1/4	187/22585	0.00535	30.194	5.335	0.032712	0.045458	0.009570	ENSG00000249915	1
CC	GO:0031461	cullin-RING ubiquitin ligase complex	1/4	195/22585	0.00513	28.955	5.218	0.034094	0.045458	0.009570	ENSG00000249915	1
CC	GO:0098562	cytoplasmic side of membrane	1/4	217/22585	0.00461	26.020	4.929	0.037885	0.047356	0.009970	ENSG00000127920	1
MF	GO:0005436	sodium:phosphate symporter activity	1/4	13/21092	0.07692	405.615	20.098	0.002463	0.033906	0.004118	ENSG00000144136	1
MF	GO:0031681	G-protein beta-subunit binding	1/4	23/21092	0.04348	229.261	15.085	0.004355	0.033906	0.004118	ENSG00000127920	1
MF	GO:0070330	aromatase activity	1/4	25/21092	0.04000	210.920	14.464	0.004733	0.033906	0.004118	ENSG00000138061	1
MF	GO:0008395	steroid hydroxylase activity	1/4	32/21092	0.03125	164.781	12.769	0.006055	0.033906	0.004118	ENSG00000138061	1
MF	GO:0043495	protein-membrane adaptor activity	1/4	40/21092	0.02500	131.825	11.406	0.007565	0.033906	0.004118	ENSG00000249915	1
MF	GO:0016712	oxidoreductase activity acting on paired donors, reduced flavin or flavoprotein as one donor	1/4	48/21092	0.02083	109.854	10.398	0.009073	0.033906	0.004118	ENSG00000138061	1
MF	GO:0016836	hydro-lyase activity	1/4	61/21092	0.01639	86.443	9.204	0.011519	0.033906	0.004118	ENSG00000138061	1
MF	GO:1990756	ubiquitin ligase-substrate adaptor activity	1/4	61/21092	0.01639	86.443	9.204	0.011519	0.033906	0.004118	ENSG00000249915	1
MF	GO:0140767	enzyme-substrate adaptor activity	1/4	73/21092	0.01370	72.233	8.396	0.013773	0.033906	0.004118	ENSG00000249915	1
MF	GO:0016835	carbon-oxygen lyase activity	1/4	79/21092	0.01266	66.747	8.063	0.014899	0.033906	0.004118	ENSG00000138061	1
MF	GO:0015370	solute:sodium symporter activity	1/4	82/21092	0.01220	64.305	7.910	0.015462	0.033906	0.004118	ENSG00000144136	1
MF	GO:0048306	calcium-dependent protein binding	1/4	83/21092	0.01205	63.530	7.861	0.015649	0.033906	0.004118	ENSG00000249915	1
MF	GO:0004497	monooxygenase activity	1/4	116/21092	0.00862	45.457	6.613	0.021820	0.042577	0.005171	ENSG00000138061	1
MF	GO:0015294	solute:monoatomic cation symporter activity	1/4	123/21092	0.00813	42.870	6.414	0.023125	0.042577	0.005171	ENSG00000144136	1
MF	GO:0015293	symporter activity	1/4	155/21092	0.00645	34.019	5.682	0.029075	0.042577	0.005171	ENSG00000144136	1
MF	GO:0020037	heme binding	1/4	156/21092	0.00641	33.801	5.663	0.029260	0.042577	0.005171	ENSG00000138061	1

ONTOLOGY	ID	Description	GeneRatio	BgRatio	RichFactor	FoldEnrich	zScore	pvalue	p.adjust	qvalue	geneID	Count
MF	GO:0005506	iron ion binding	1/4	164/21092	0.00610	32.152	5.516	0.030743	0.042577	0.005171	ENSG00000138061	1
MF	GO:0015081	sodium ion transmembrane transporter activity	1/4	164/21092	0.00610	32.152	5.516	0.030743	0.042577	0.005171	ENSG00000144136	1
MF	GO:0046906	tetrapyrrole binding	1/4	166/21092	0.00602	31.765	5.481	0.031114	0.042577	0.005171	ENSG00000138061	1
MF	GO:0016705	oxidoreductase activity acting on paired donors with incorporation or reduction of molecular oxygen	1/4	190/21092	0.00526	27.753	5.102	0.035551	0.046216	0.005613	ENSG00000138061	1
MF	GO:0016829	lyase activity	1/4	216/21092	0.00463	24.412	4.763	0.040341	0.049946	0.006066	ENSG00000138061	1

Table S5. Summary of experimental design.

Assay / Experiment	Cell model	Radiation dose (Gy)	Tameron® concentration range	Pretreatment duration	Post-irradiation time point	Biological replicates	Primary endpoint
H_2O_2 generation in aqueous solution	Cell-free (buffer)	5 and 15 Gy	0, 0.5, 1, 5 mM	Drug added before irradiation	Measured at time of irradiation	n = 3 independent experiments	H_2O_2 concentration (nM)
MTT assay (metabolic activity)	hMSC and MNNG/Hos	0 and 15 Gy	0.25–4 mM	6 h before irradiation	72 h post-irradiation	n = 3 (SD shown)	Metabolic activity (% of control)
Cell counting (hMSC) / Clonogenic assay (MNNG/Hos)	hMSC and MNNG/Hos	0 and 5 Gy	0.25–4 mM (cell count); 0.25–2 mM (clonogenic)	Before irradiation (overnight culture + drug)	8 days post-irradiation	n = 5 (SD shown)	Cell number (*10^3); colony count (% of irradiated control)
Live/Dead assay	hMSC and MNNG/Hos	0 and 15 Gy	0.25–4 mM	Before irradiation	Fixed after irradiation (single time point)	n = 6	% live and dead cells
Intracellular ROS — CellROX (peroxides) and DHE (superoxide)	hMSC and MNNG/Hos	15 Gy	0.25–2 mM	3 h before irradiation	Continuous, 0–20 h post-irradiation	n = 8 wells/group	Fluorescence intensity (a.u.)
Non-enzymatic antioxidants — ThiolTracker (reduced glutathione)	hMSC and MNNG/Hos	15 Gy	0.25–2 mM	3 h before irradiation	Continuous, 0–20 h post-irradiation	n = 8 wells/group	Fluorescence intensity (a.u.)
DNA double-strand breaks — γH2AX foci	hMSC and MNNG/Hos	1.5 Gy	0.25, 0.5, and 1 mM	24 h before irradiation	1 h and 4 h post-irradiation	n = 30 cells/group (≥20 fields)	γH2AX foci per cell
Transcriptomic profiling — nanopore sequencing	MNNG/Hos	0 and 15 Gy	0.25 mM and 2 mM	Before irradiation (groups per Table 1)	Day 1 and Day 3 post-treatment	Per experimental group (Table 1)	Differentially expressed genes; pathway enrichment

Abbreviations: hMSC, human mesenchymal stem cells; MNNG/Hos, human osteosarcoma cell line; DHE, dihydroethidium; ROS, reactive oxygen species; DSB, DNA double-strand breaks; a.u., arbitrary units; SD, standard deviation.
